# Meta-analysis shows that mesenchymal stem cell therapy can be a possible treatment for diabetes

**DOI:** 10.3389/fendo.2024.1380443

**Published:** 2024-05-10

**Authors:** Umm E. Habiba, Nasar Khan, David Lawrence Greene, Khalil Ahmad, Sabiha Shamim, Amna Umer

**Affiliations:** ^1^Research and Development (R&D) Department, R3 Medical Research LLC, Scottsdale, AZ, United States; ^2^Research and Development (R&D) Department, Pak-American Hospital Pvt. Ltd., Islamabad, Pakistan; ^3^Research and Development (R&D) Department, R3 Stem Cell LLC, Scottsdale, AZ, United States; ^4^Research and Development (R&D) Department, Bello Bio Labs and Therapeutics Pvt. Ltd., Islamabad, Pakistan; ^5^Department of Statistics, Quaid-i-Azam University, Islamabad, Pakistan

**Keywords:** diabetes mellitus, mesenchymal stem cell, stem cell therapy, regenerative medicine, clinical trials

## Abstract

**Objective:**

This meta-analysis includes the systematic literature review and meta-analysis involving clinical trials to assess the efficacy and safety of mesenchymal stem cell (MSC) transplantation for treating T1DM and T2DM.

**Methods:**

We searched PubMed, ScienceDirect, Web of Science, clinicaltrials.gov, and Cochrane Library for “published” research from their inception until November 2023. Two researchers independently reviewed the studies’ inclusion and exclusion criteria. Our meta-analysis included 13 studies on MSC treatment for diabetes.

**Results:**

The MSC-treated group had a significantly lower HbA1c at the last follow-up compared to the baseline (MD: 0.95, 95% CI: 0.33 to 1.57, *P*-value: 0.003< 0.05), their insulin requirement was significantly lower (MD: 0.19, 95% CI: 0.07 to 0.31, *P*-value: 0.002< 0.05), the level of FBG with MSC transplantation significantly dropped compared to baseline (MD: 1.78, 95% CI: -1.02 to 4.58, *P*-value: 0.212), the FPG level of the MSC-treated group was significantly lower (MD: -0.77, 95% CI: -2.36 to 0.81, *P*-value: 0.339 > 0.05), and the fasting C-peptide level of the MSC-treated group was slightly high (MD: -0.02, 95% CI: -0.07 to 0.02, *P-*value: 0.231 > 0.05).

**Conclusion:**

The transplantation of MSCs has been found to positively impact both types of diabetes mellitus without signs of apparent adverse effects.

## Introduction

Diabetes is a serious and growing health problem worldwide. This is a persistent health condition that arises when the body is incapable of adequately controlling the levels of glucose in the bloodstream. Each year, the prevalence of diabetes mellitus (DM) rises. According to the International Diabetes Federation, approximately 4.51 million adults across the globe were diagnosed with diabetes in 2017. Furthermore, it is expected that this figure will escalate to 6.93 million by the year 2045 (1). Diabetes holds two types which vary in their mode of action on the human body. Type 1 diabetes mellitus (T1DM) is an immune system infection, and insusceptible assaults result in the obliteration of islet cells, causing islet aggravation related to outright insulin lack. Eventually, several related complications arise, compromising the patient’s quality of life and reducing their durability (2). Around 90% of adults with diabetes are diagnosed with the most common form of diabetes, known as type 2 diabetes mellitus (T2DM) (3). The leading causes of diabetes are the malfunctioning of islet cells and the body’s reduced sensitivity to insulin (4).

High blood glucose levels in individuals with diabetes are managed through a combination of insulin injections, daily oral hypoglycemic agents, exercise, and diet. However, while these conventional therapeutic approaches aim to regulate insulin levels, they may not always be effective in doing so, which can result in severe hypoglycemia and poor adherence to treatment plans. In fact, only 14% of patients with diabetes in the United States meet the glucose, lipid, and blood pressure control and quitting smoking targets. Despite significant research efforts devoted to understanding the disease process and the experimental therapeutics of diabetes, there is still an urgent need for more effective treatments to prevent or manage this severe metabolic illness (5).

Stem cell-based transplantation has emerged as a promising strategy for treating diabetes in recent years, which offers numerous benefits. Mesenchymal stem cells (MSCs), unlike embryonic stem cells, are not associated with tumorigenic risks or ethical concerns when treating diabetes (6–8). Due to its ease of access and wide availability, MSC transplantation is an appealing option (9); it has low immunogenicity, the ability to self-renew, the potential for multi-differentiation, the secretion of various cytokines, and other biological characteristics. It does not raise any ethical issues (10–12).

In the past decade, MSCs have demonstrated their therapeutic potential in both clinical and preclinical studies for the treatment of diabetes. *In vitro* studies have proposed that MSCs are capable to self-renew and differentiate into multiple mesenchymal lineages such as adipogenic, chondrogenic, and osteogenic lineages. Furthermore, they have low immunogenicity due to the interstitial expression of major histocompatibility complex (MHC) class I and the lack of MHC class II. MSCs release cytokines, growth factors, and exosomes, which modulate insulin sensitivity and β-cell dysfunction. Earlier studies have recommended that MSCs have the capability to exert antidiabetic effects because several dose administrations of MSCs may help improve hyperglycemia in DM patients.

Recent experimental explorations shed light on the complex mechanisms that highlight the therapeutic effects of MSCs in diabetes management. In STZ diabetic animal models, β-cell dysfunction can be caused by pancreatic microenvironment inflammation. The MSC treatment has been validated to facilitate the proliferation of regulatory T cells (Tregs) and incorporate long-term immunomodulatory effects. The secretion of cytokines such as Th2 secreted by Tregs and interleukins (IL-10 and IL-3) pose an anti-inflammatory profile which supports pancreatic β-cell regeneration and function (13).

In addition, MSCs exhibit a response toward inflammatory stimuli by shifting macrophages from pro-inflammatory (M1) to anti-inflammatory (M2) phenotypes. This is promoted by an overexpression of IL-6 and monocyte chemoattractant protein (MCP-1). MSCs may alleviate the systemic inflammation by downregulating the inflammatory cytokines, reducing insulin receptor action, and secreting IL-1Ra in response to IL-1β and tumor necrosis factor (TNF-α) signals from diabetic islets. This reaction reduces the synthesis of NLRP3 production in adipose tissue and liver. These findings highlight MSCs’ complex immunomodulatory characteristics and potential as a therapeutic method for controlling type 2 diabetes and its consequences. Conclusively, MSC infusion has been utilized to treat diabetes by reconstructing β cells, enhancing and regulating glucose homeostasis, alleviation of insulin resistance, and lowering/regulating systemic inflammation (14).

The therapeutic potential of bone marrow-obtained mesenchymal stem cell (BM-MSC) transplantation in treating T2DM was demonstrated for the first time in a 2009 study conducted by Bhansali et al. (15). The study involved 10 T2DM patients and showed that BM-MSC transplantation developed a significant decline in insulin requirement and improvement in stimulated C-peptide levels. Several subsequent studies have been carried out to validate the safety and effectiveness of using BM-MSCs and placenta-derived mesenchymal stem cells (PD-MSCs) in treating T2DM. These investigations have confirmed the initial results reported in (15) and have provided further evidence of the therapeutic potential of BM-MSCs and PD-MSCs for treating T2DM (16).

A recent investigation demonstrated that patients with T2DM experienced a decrease in HbA1c levels and insulin dose at the 6-month mark following treatment and after receiving a combination of intravenous and intrapancreatic endovascular injection of umbilical cord-derived mesenchymal stem cells (UC-MSCs) with a 5-day interval. The study also discovered that 41% of the patients became insulin independent, and 29% had 50% or greater reduction in insulin requirement. Nonetheless, these positive outcomes were not sustained over the next 3–6 months as the HbA1c levels and insulin dose reverted to their pre-treatment levels (17).

Multipotent stem cells have been utilized in the treatment of different autoimmune-related disorders, with some commercial products resulting from these treatments (18–21). However, the assessment of the safety and effectiveness of stem cell transplantation for DM is still in its preliminary stages. Clinical trials involving MSCs and hematopoietic stem cells (HSCs) in patients with T1DM have been carried out since 2000 (22–25), yet there is still no strong consensus on their efficacy. To date, no research has been carried out to compare the effectiveness of mesenchymal stem cells (MSCs) in treating type 1 diabetes mellitus (T1DM) and type 2 diabetes mellitus (T2DM) despite earlier studies demonstrating the efficacy of MSCs in both forms of diabetes. Furthermore, a number of studies have been published in the context of systematic reviews and meta-analyses. They examined the impact of stem cell therapy (SCT) on diabetes mellitus, but the absence of several critical components in each of these investigations has led to differences in their findings and has resulted in them being inadequate in providing a complete understanding of previous interventional studies.

Recently, Madani et al. (26) compared the efficacy of MSCs and HSCs in a meta-analysis of SCT research in T1DM. The search period for this paper was limited to September 2019, and it does not include both types of DM to compare the effectiveness of MSCs and HSCs simultaneously. Similarly, a meta-analysis (27) was carried out in 2021 to predict the safety and efficacy profile of transplanting mesenchymal stem cells for treating T1DM and T2DM. However, this meta-analysis did not include four papers (28–31) as their search window was limited to November 2011 to November 2020. The reason of not including these studies is not mentioned. Our meta-analysis includes the recent clinical trial conducted by Zang et al. (32), which involved testing UC-MSCs on Chinese adults with T2DM. The primary objective of this study was to evaluate the distinct therapeutic effects of MSCs on diabetes mellitus and its subtypes as well as their safety to lay a speculative foundation for medical assessment and diabetes therapeutic interventions based on trials conducted until November 2023. The outcomes of this research could have the potential to guide in the design of future clinical trials investigating the effectiveness and safety of MSC therapy for DM as well as provide evidence to support the development of clinical guidelines for the use of this therapy.

## Materials and methods

### Selection criteria

The studies were included on the following criteria: (1) research studies published in Chinese and English, (2) clinical studies/trials involving MSCs as a treatment regimen for DM, (3) MSCs were used to treat diabetes in all patients, regardless of age, race, sex, extent of disease, or geographical location, and (4) all findings evaluated the treatment of diabetes with MSCs. There were no restrictions on the time, duration, or dosing frequency of MSCs used in the treatments. The treatment’s control group is either placebo or absolutely nothing. The treatment duration and dosage for the placebo was the same as for the MSC group. Finally, these are (5) studies with multiple follow-up timeframes, ranging from 3-, 6-, 9-, and 12 months, which was consistent with the majority of the studies analyzed.

The exclusion criteria were (1) research studies/trials in languages apart from Chinese and English, (2) studies with lacking reports or data (such as conference abstracts with missing sections), and (3) repeat publications. The meta-analysis incorporated the most up-to-date and comprehensive studies available, including clinical trials.

### Outcomes

The study looked at two types of outcomes:

1. **Primary outcomes:** These were variations in insulin requirements, HbA1c, fasting blood glucose (FBG), fasting plasma glucose (FPG), and C-peptide between baseline and after therapy (3-, 6-, 9-, and 12-month follow-up).
2. **Secondary outcomes:** These included hypoglycemia episodes, self-limiting upper respiratory tract infections, mild fever, nausea, and vomiting.

### Search strategy

We started searching numerous directories for eligible studies, including the Cochrane Library, PubMed, ScienceDirect, Web of Science, and clinicaltrials.gov while following the PRISMA 2020 guidelines. We used a combination of keywords, including (“mesenchymal stem/stromal cell, Wharton’s jelly cells, progenitor cells, bone marrow” or “MSCs”) AND (“diabetic, diabetes mellitus or hyperglycemia”) AND (“type 1 diabetes” or “type 2 diabetes”) AND “clinical trial” AND (“English language” OR “Chinese language”). In addition to database searches, we also performed manual searches of reference lists and descriptive reviews from applicable trials. The exploration was strictly limited to human subjects, published studies, case studies, and English and Chinese papers, with unpublished studies being excluded. The search period covered all publications up to February 2023. A detailed description of the search strategies is provided in Appendix 1.

### Data extraction and basic characteristics

Two researchers (DLG and SS) worked independently on the comprehensive literature screening and data retrieval. In cases where discrepancies arose during the study selection process, a third reviewer was consulted. We collected pertinent information for all selected studies, such as the first author’s name, year of publication, sample size, study type, mean patient age in years, mean dose of injected cells, treatment route, number of patients who achieved insulin-free status, and timeframe of follow-up duration in months (**Table 1**).

**Table 1:** Baseline characteristics of 13 eligible papers included in this meta-analysis.

References	Sample size	Study type	Type of diabetes	Treatment	Mean age of patients (years)	Mean dose of injected cells	Route of injection	Number of insulin free	Mean follow-up period (months)
Bhansali et al. (15)	10	n-RCT	T2DM	BM-MSCs	57.5 ± 5.9	3.5 ± 1.4 × 10^8^	Transfemoral route into gastroduodenal artery beyond the origin of cystic artery	0	6
Jiang et al. (16)	10	Pilot study	T2DM	PD-MSCs	66	1.35 × 10^6^	IV	0	= 3
Yu et al. (33)	12	RCT	TIDM	hUC-MSCs	19.67 ± 2.58 14.83 ± 8.18	1 × 10^7^	IV	0	9
Hu et al. (34)	29	RCT	TIDM	WJ-MSCs	17.6	2.6 × 10^7^	Peripheral vein	3	24
Mesples et al. (30)	3	Case study	TIDM	BM-MSCs	7	181 × 10^6^	Intra-hepatic parenchyma	0	12
Liu et al. (17)	22	Prospective, non-placebo	T2DM	WJ-MSCs	52.9 ± 10.5	1 × 10^6^	Spleen artery	0	12
Carlsson et al. (35)	18	RCT	TIDM	BM-MSC; Control	24 ± 6 27 ± 6	2.75 × 10^6^	Peripheral vein	0	12
Guan et al. (36)	6	n-RCT	T2DM	hUC-MSCs	40.5 ± 3.76	(0.88 ± 0.05) × 10^6^	Elbow vein	3	33.2 ± 2.82
Esfahani et al. (28)	23	n-RCT	TIDM	BM-MSCs	12.56	2 × 10^6^	Peripheral vein	2	12
Hu et al. (37)	61	RCT	T2DM	WJ-MSCs	52.43 ± 4.88 53.21 ± 8.22	1 × 10^6^	IV	6	36
Bhansali et al. (38)	30	RCT	T2DM	ABM-MSCs; ABM-MNCs; control	47.9 ± 18.9 44.6 ± 8.9 51.7 ± 13.3	(1.2 ± 0.3) × 10^9^	SPD artery; splenic artery; transfemoral route into the femoral artery	0	12
Ulyanova et al. (31)	5	Case study	TIDM	AMSCT	30	96 × 10^6^	Peripheral vein	0	3
Zang et al. (32)	73	RCT	T2DM	UC-MSCs	50.00 + 9.38 50.45 + 8.03	1 × 10^6^	Elbow joint (IV)	5	12

RCT, randomized controlled trial; n-RCT, non-randomized controlled trial; BM-MSCs, bone marrow-derived mesenchymal stem cells; PD-MSCs, placenta-derived mesenchymal stem cells; hUC-MSCs, human umbilical cord-derived mesenchymal stem cells; ASC, amniotic stem cell transplantation; WJ-MSCs, Wharton’s jelly-derived mesenchymal stem cells; ABM-MSCs, autologous bone marrow-derived mesenchymal stem cells; ABM-MNCs, autologous bone marrow-derived mononuclear cells; SPD, superior pancreatico-duodenal; IV, intravenous; AMSCT autologous mesenchymal stem cell transplantation.

### Statistical data analysis

This analysis utilized mean difference (MD) to compare continuous variables between baseline and follow-ups. MD was selected to compare continuous variables because of its simplicity, interpretability, and compatibility with meta-analysis techniques, representing the absolute difference in means between treatment groups, quantifying the magnitude of difference, and providing a straightforward measure of treatment effect. It is suitable for synthesizing data from diverse studies, focusing on comparing means rather than specific statistical assumptions, and allows to estimate the overall effect. We considered *P*-value less than 0.05 with a 95% confidence interval (CI) as statistically significant. We calculated the heterogeneity of the included studies using the *I*^2^ statistic, where values of 25%, 50%, and 75%–100% indicated low, medium, and high heterogeneity, respectively. In instances where significant heterogeneity was detected (*I*^2^ > 50% and *P*< 0.10), we used a random-effects model for the meta-analysis (39). Otherwise, the data were evaluated using a fixed-effects model.

These models offer the flexibility to incorporate prior information and estimate heterogeneity more comprehensively. The decision to utilize fixed-effects or random-effects models in our meta-analysis was indeed based on the observed level of heterogeneity among the included studies. While fixed-effects models assume a common treatment effect across all studies, random-effects models account for both within-study and between-study variability, acknowledging potential differences in treatment effects. Moreover, in case of higher heterogeneity, the Knapp and Hartung adjustment was also applied. This adjustment accounts for potential variability in effect sizes across studies and provides more conservative estimates of the overall effect. We also performed sensitivity analysis to evaluate the robustness of results by testing the impact of different assumptions, models, or inclusion criteria, thus ensuring the reliability and validity of conclusions amidst varying methodological choices and potential biases. The externally standardized residuals, DFFITS values, Cook’s distances, covariances ratios, leave-one-out Tau estimates, Hat values, and weights were plotted. This allows for a comprehensive assessment of the data and helps identify influential data points or outliers. This approach enhances the transparency and reliability of the analysis. In this meta-analysis, we compared the treatment group, i.e., MSCs and the control group (if any) from the selected studies using Jamovi version 2.3 (40), and the results were depicted by forest plots (**Tables 2**–**6**). We assessed the heterogeneity and publication bias using several methods, including the Q Cochrane test and *I*^2^ statistic, the Cochrane ROB tool, meta-regression analysis, and examination of publication bias using funnel plots, and Begg’s and Egger’s regression tests.

**Table 2:** Forest plots with the corresponding 95% CIs for the mean difference (MD) of HbA1c.

	Statistics	Forest plot Studies MD [95% CI] Weight (%) MD [95% CI]	Funnel plot
3-month follow-up Heterogeneity		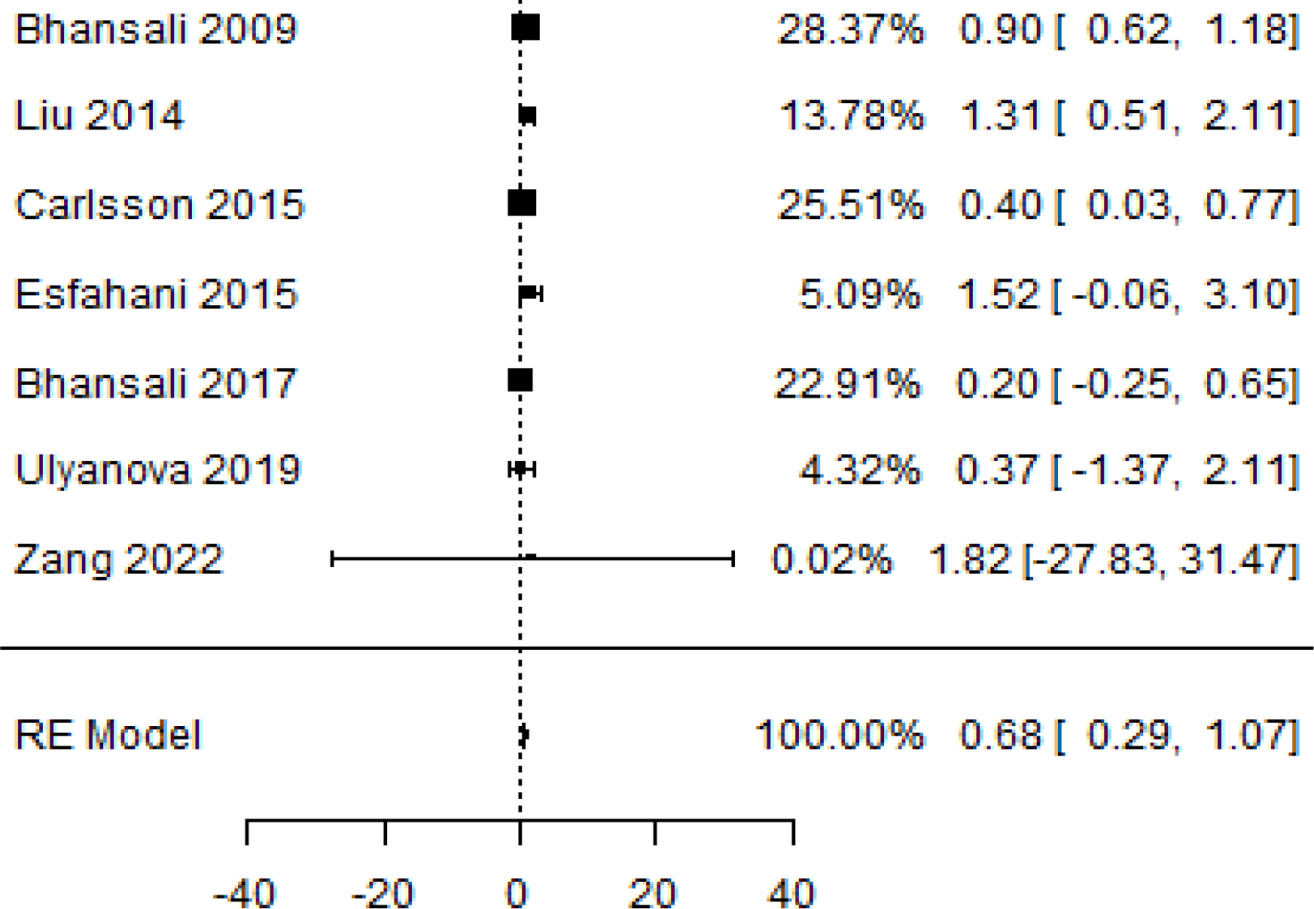	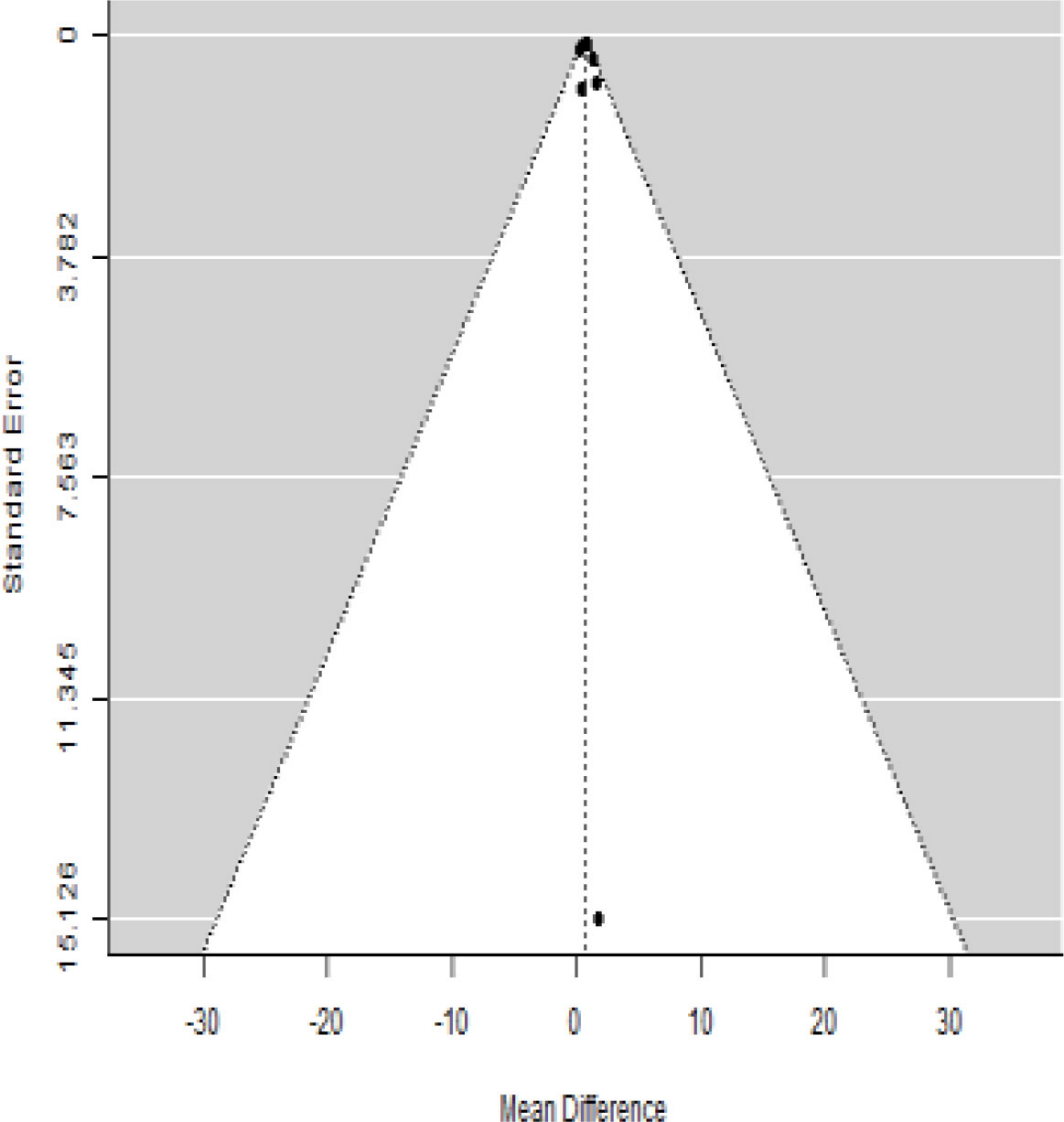
$Tau^{2}$ ( $\tau^{2}$ )	0.1184		
$I^{2}$	58.33%		
d.f	6.000		
Q-test ( $\chi^{2}$ )	12.547		
P−value	0.0		
Test for overall effect			
Z−test	3.42		
P−value	< 0.001		
Publication bias assessment			
Begg and Mazumdar ( P−value )	0.773		
Egger’s regression ( P−value )	0.568		
6-month follow-up Heterogeneity		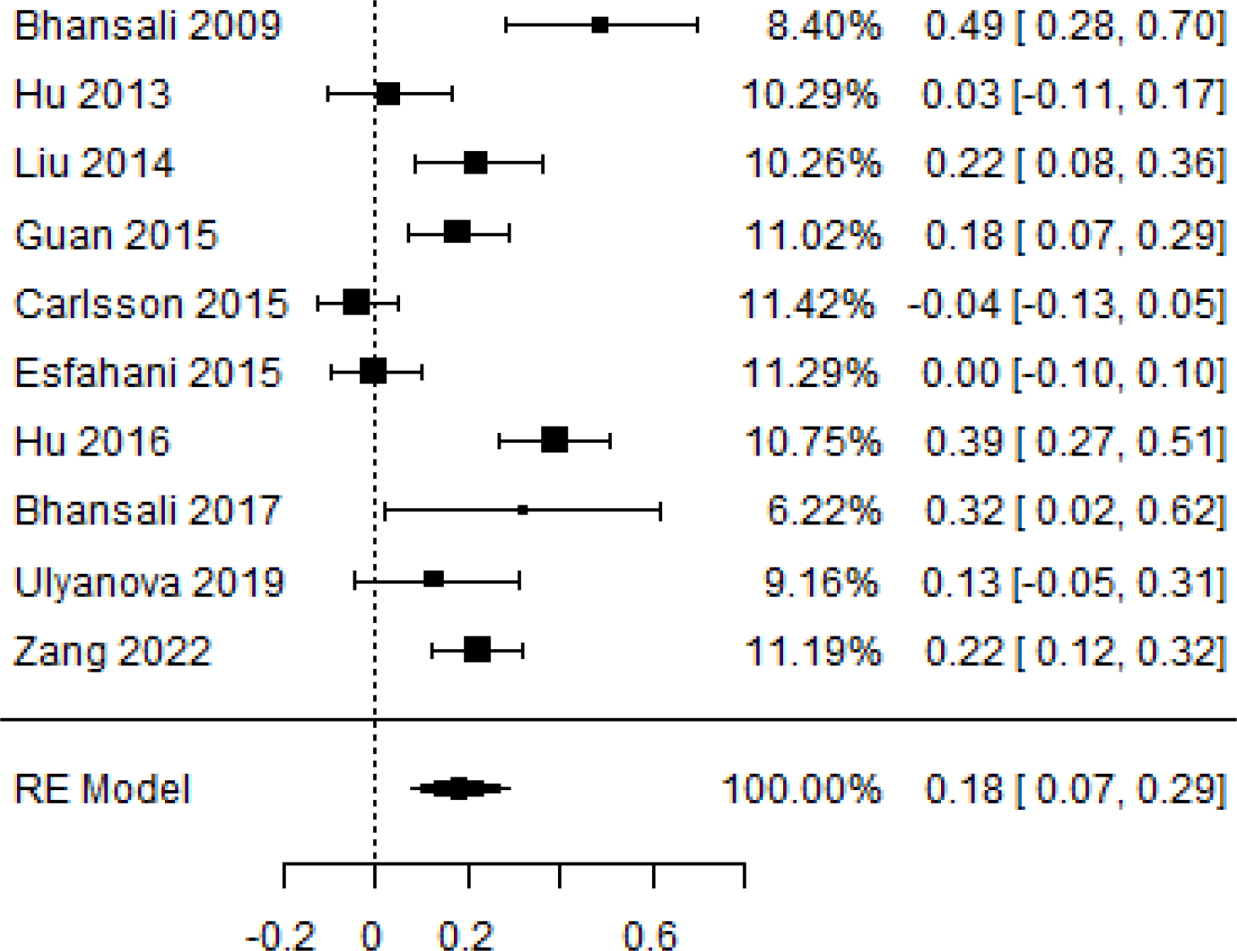	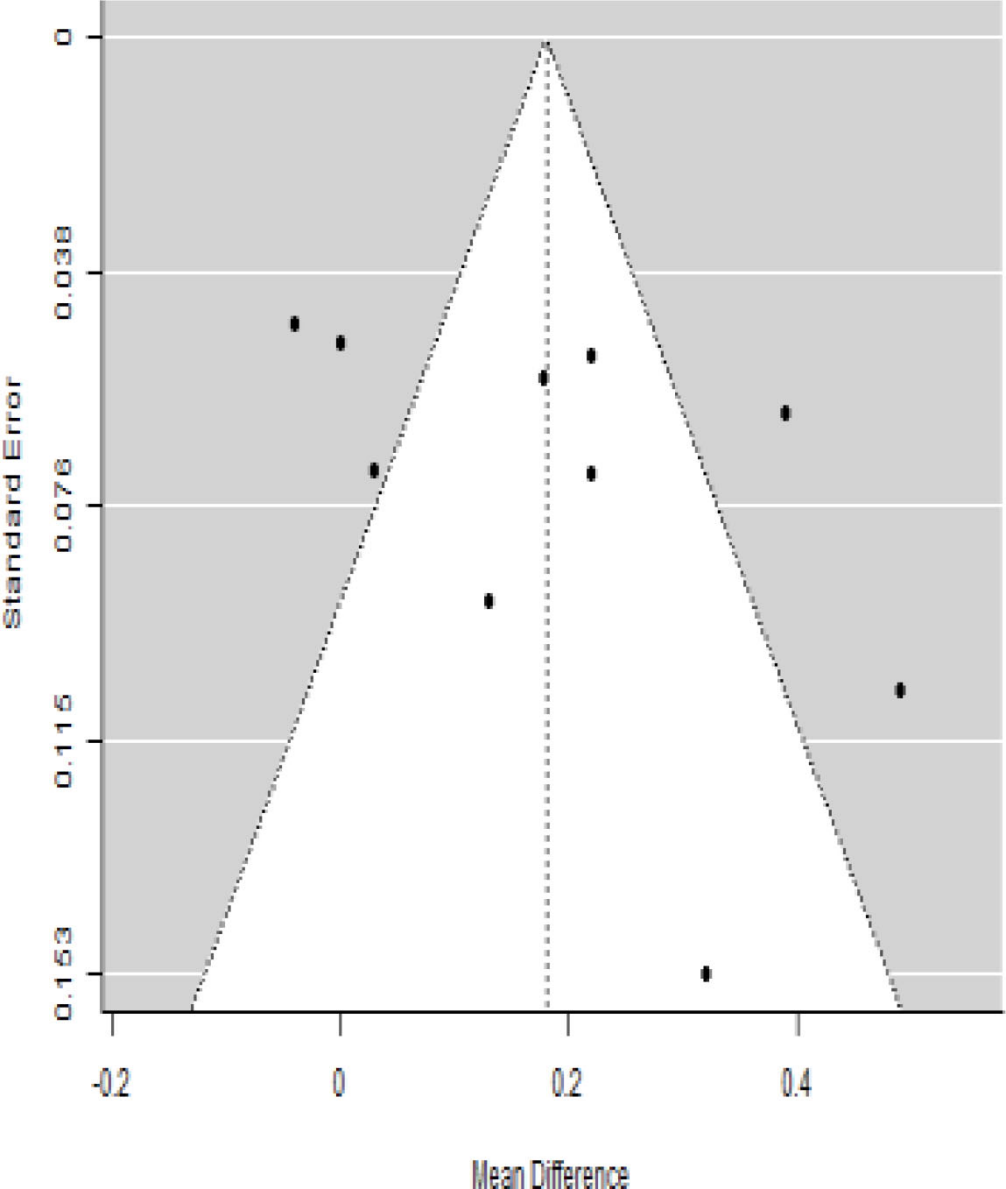
$Tau^{2}$ ( $\tau^{2}$ )	0.0233		
$I^{2}$	84.84%		
d.f	9.000		
Q-test ( $\chi^{2}$ )	58.590		
P−value	<.001		
Test for overall effect			
Z−test	3.35		
P−value	< 0.001		
Publication bias assessment			
Begg and Mazumdar ( P−value )	0.156		
Egger’s regression ( P−value )	0.091		
9-month follow-up Heterogeneity		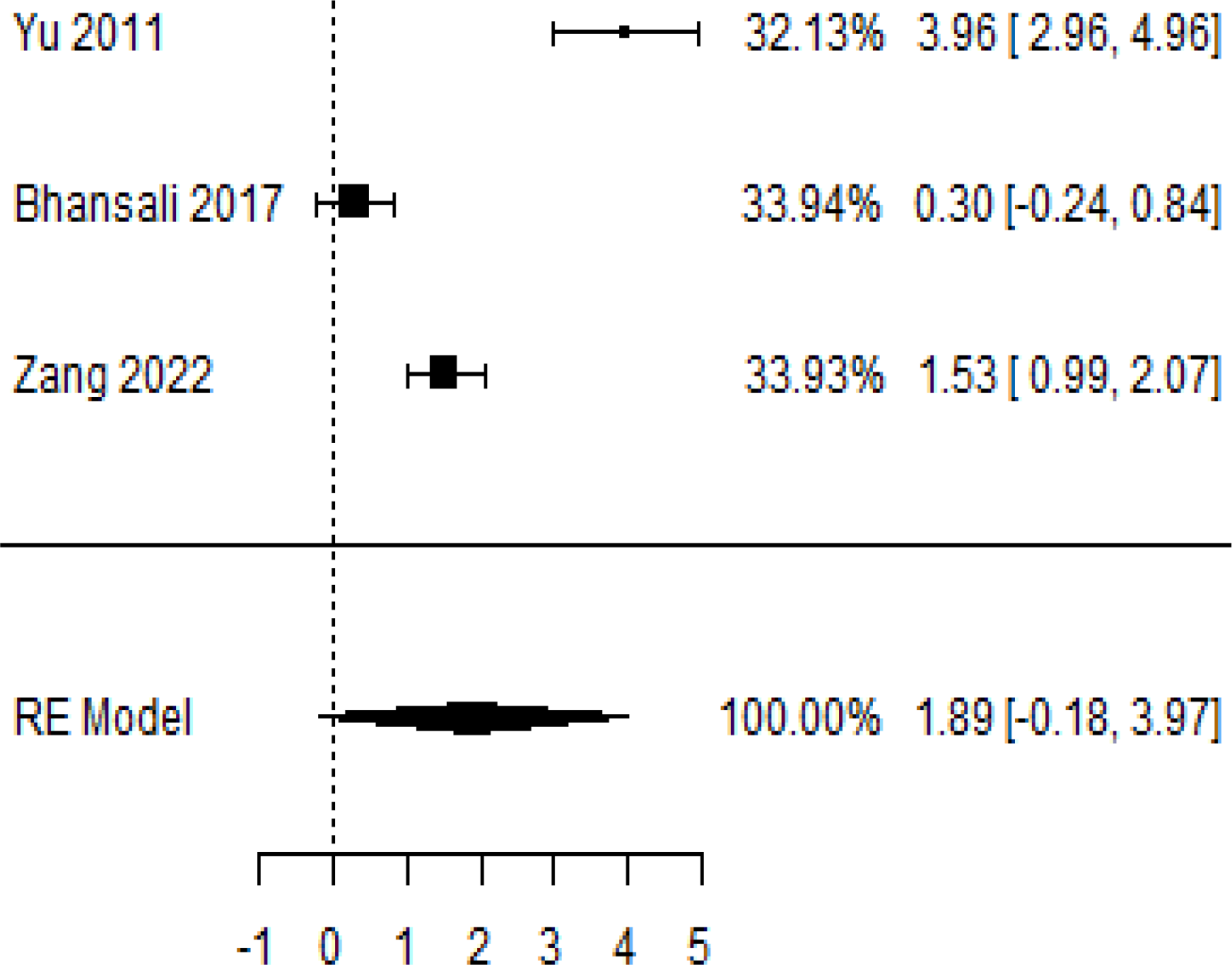	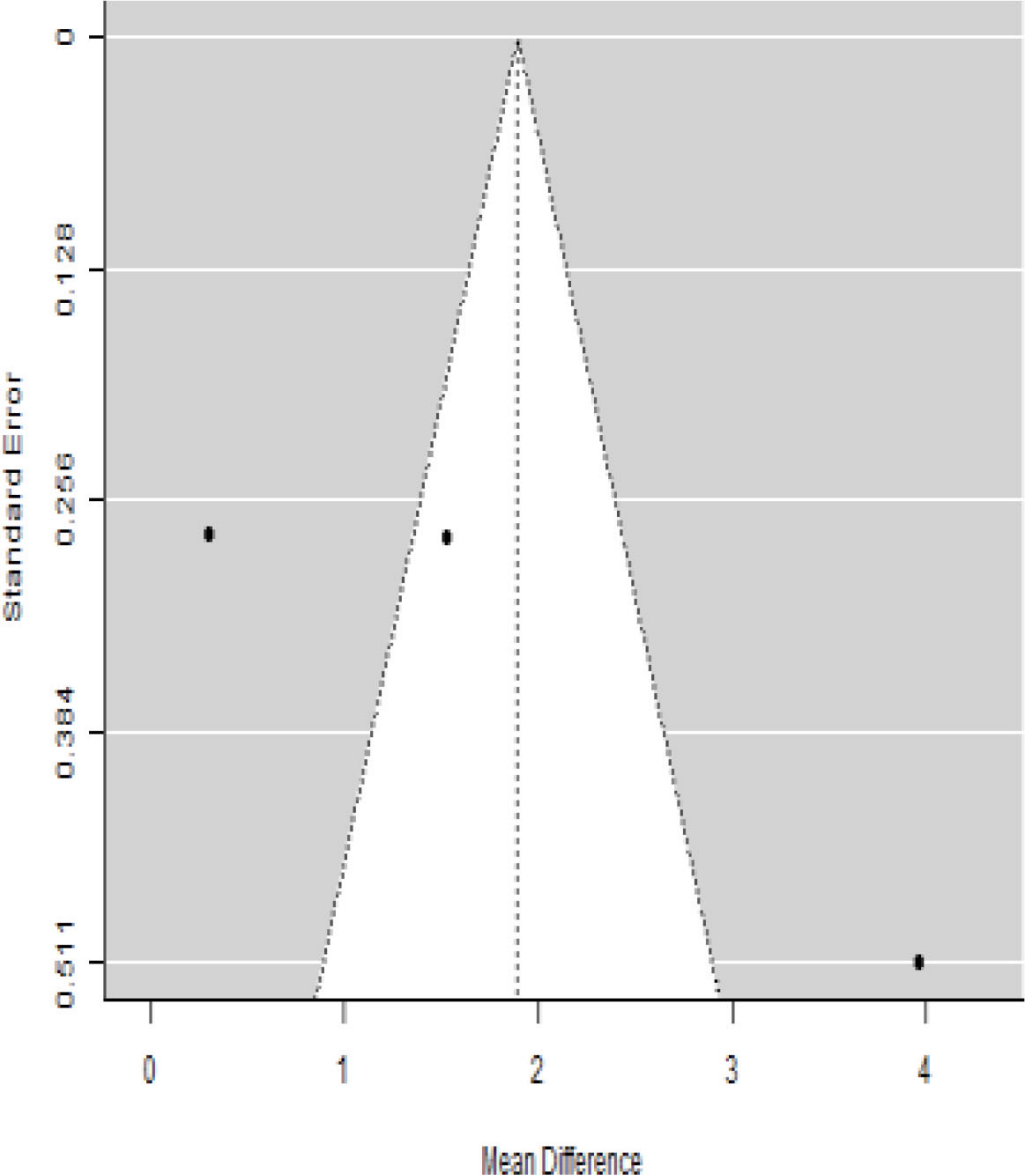
$Tau^{2}$ ( $\tau^{2}$ )	3.2342		
$I^{2}$	96.73%		
d.f	2.000		
Q-test ( $\chi^{2}$ )	41.073		
P−value	< 0.001		
Test for overall effect			
Z−test	1.79		
P−value	0.074		
Publication bias assessment			
Begg and Mazumdar ( P−value )	0.333		
Egger’s regression ( P−value )	0.007		
12-month follow-up Heterogeneity		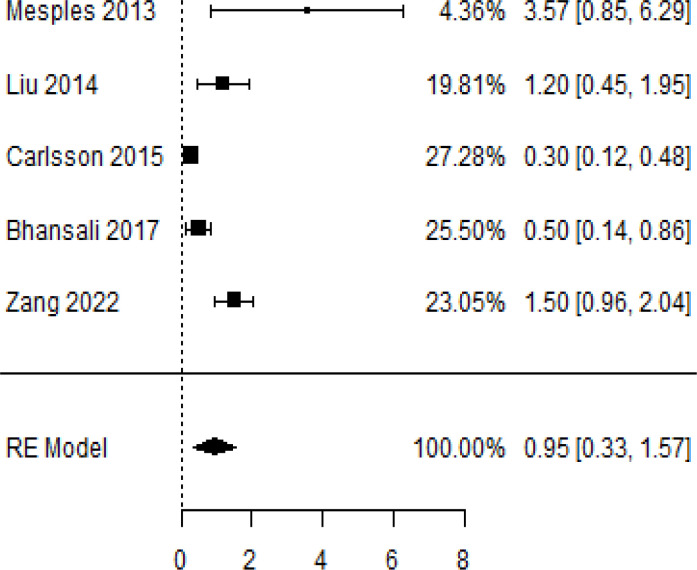	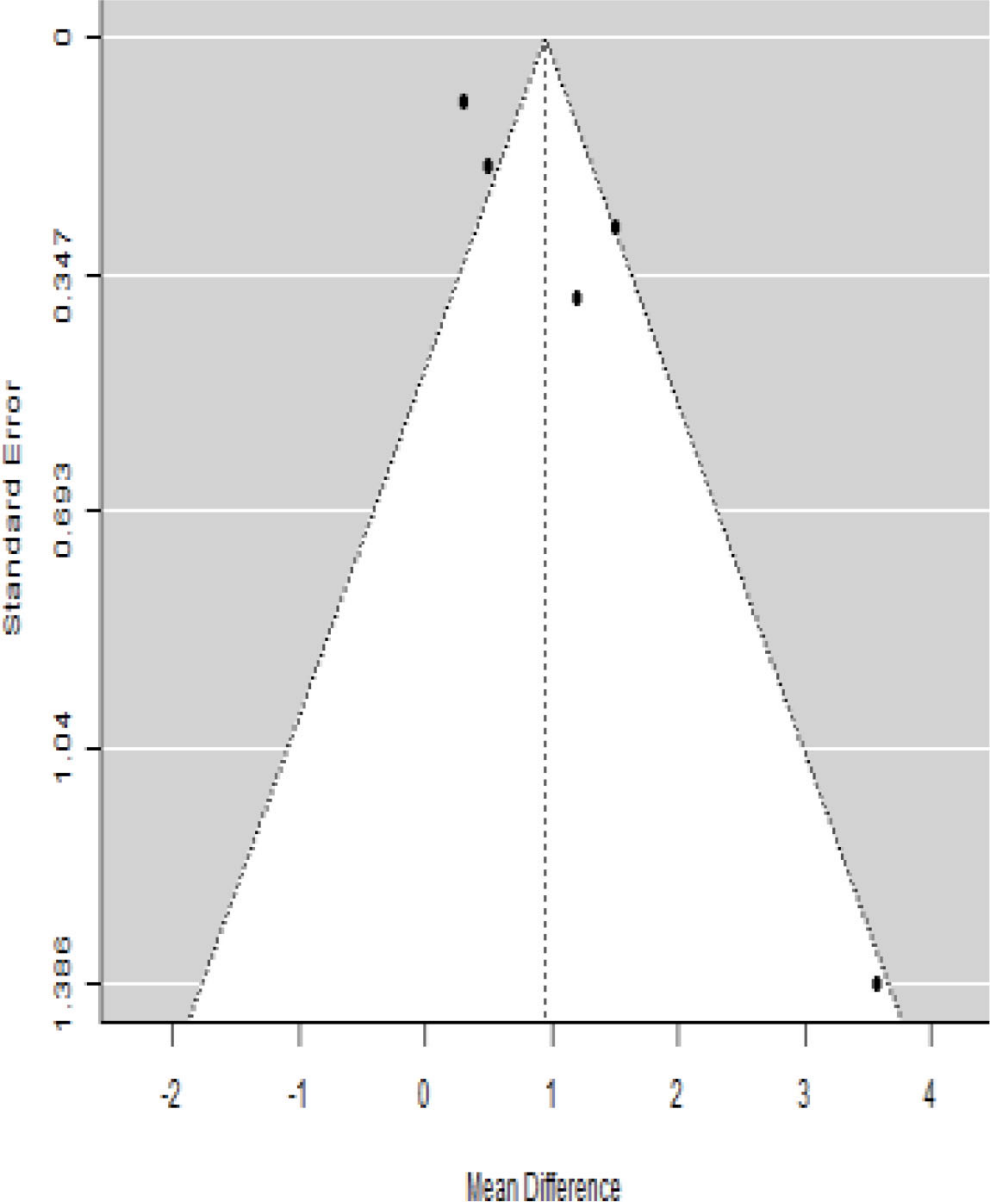
$Tau^{2}$ ( $\tau^{2}$ )	0.3551		
$I^{2}$	87.33%		
d.f	4.000		
Q-test ( $\chi^{2}$ )	25.908		
P−value	< 0.001		
Test for overall effect			
Z−test	3.01		
P−value	0.003		
Publication bias assessment			
Begg and Mazumdar ( P−value )	0.233		
Egger’s regression ( P−value )	0.001		

**Table 3:** Forest plots with the corresponding 95% CIs for the mean difference (MD) of insulin requirement.

	Statistics	Forest plot Studies MD [95% CI] Weight (%) MD [95% CI]	Funnel plot
3-month follow-up Heterogeneity		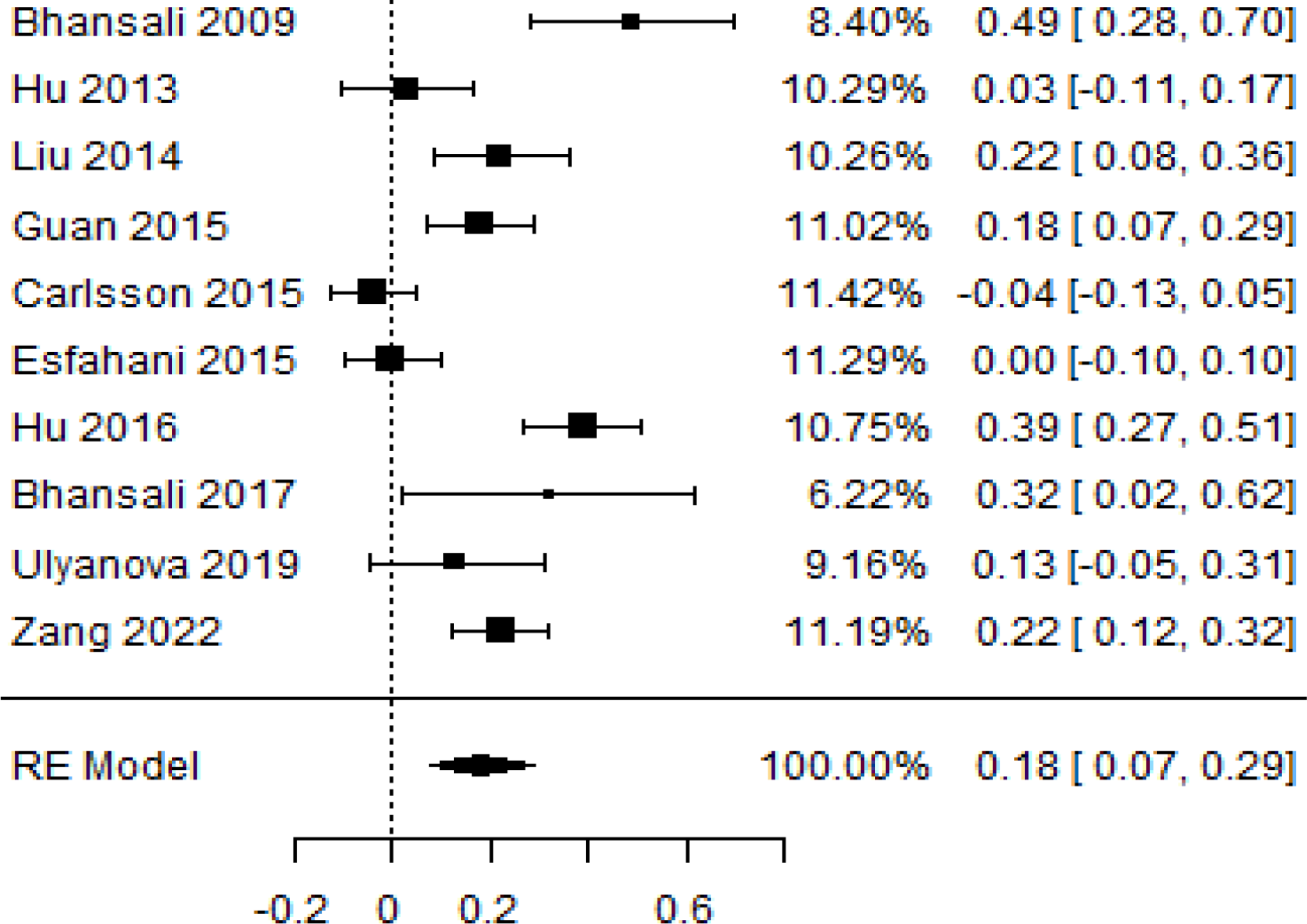	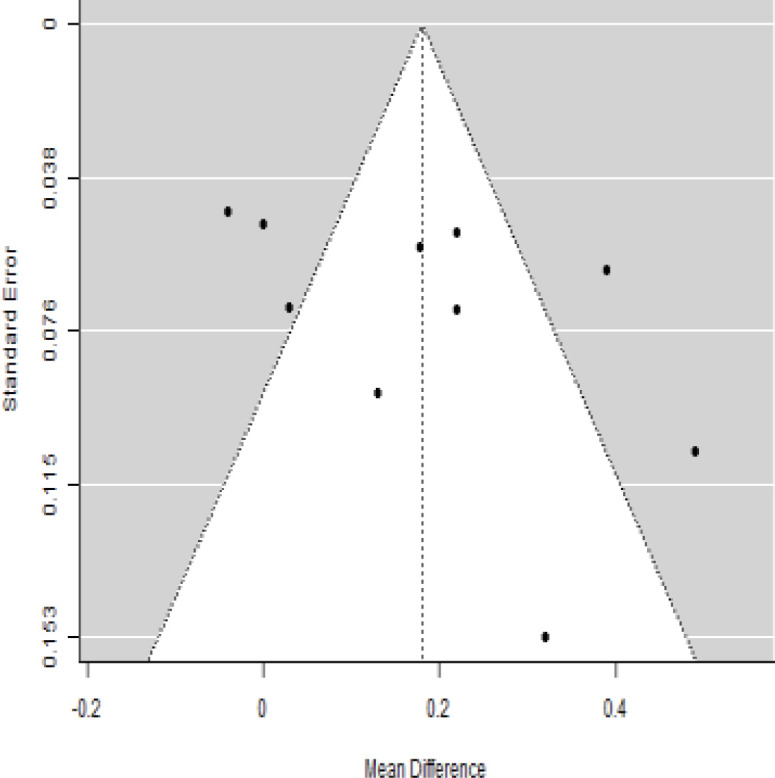
$Tau^{2}$ ( $\tau^{2}$ )	0.0233		
$I^{2}$	84.84%		
d.f	9.000		
Q-test ( $\chi^{2}$ )	58.590		
P−value	< 0.001		
Test for overall effect			
Z−test	3.35		
P−value	< 0.001		
Publication bias assessment			
Begg and Mazumdar ( P−value )	0.156		
Egger’s regression ( P−value )	0.091		
6-month follow-up Heterogeneity		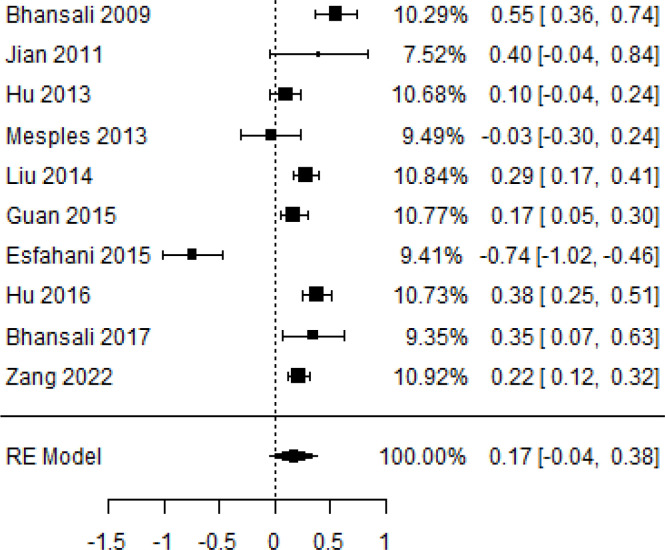	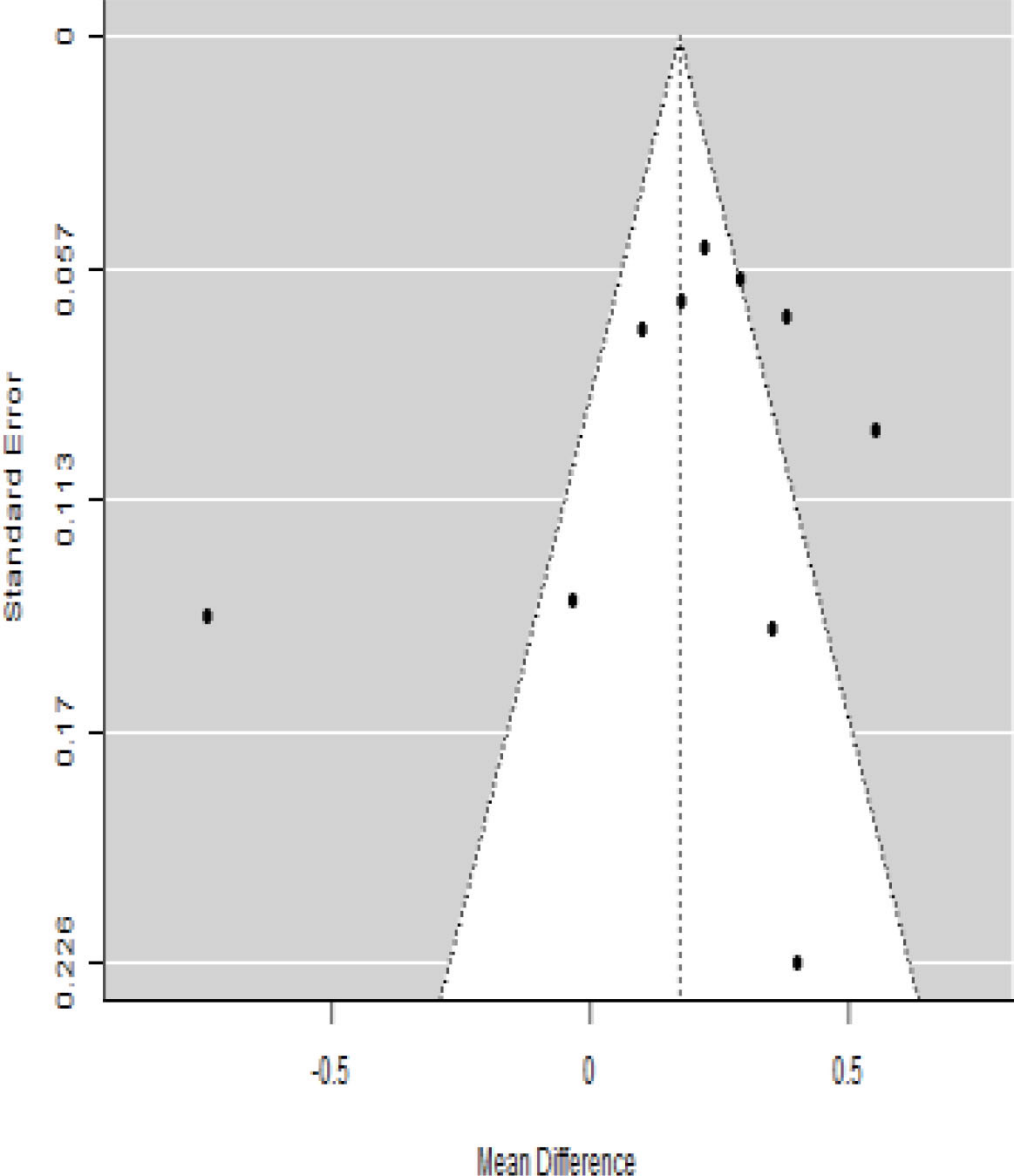
$Tau^{2}$ ( $\tau^{2}$ )	0.105		
$I^{2}$	94.02%		
d.f	9.000		
Q-test ( $\chi^{2}$ )	72.793		
P−value	< 0.001		
Test for overall effect			
Z−test	1.59		
P−value	0.112		
Publication bias assessment			
Begg and Mazumdar ( P−value )	0.601		
Egger’s regression ( P−value )	0.619		
9-month follow-up Heterogeneity		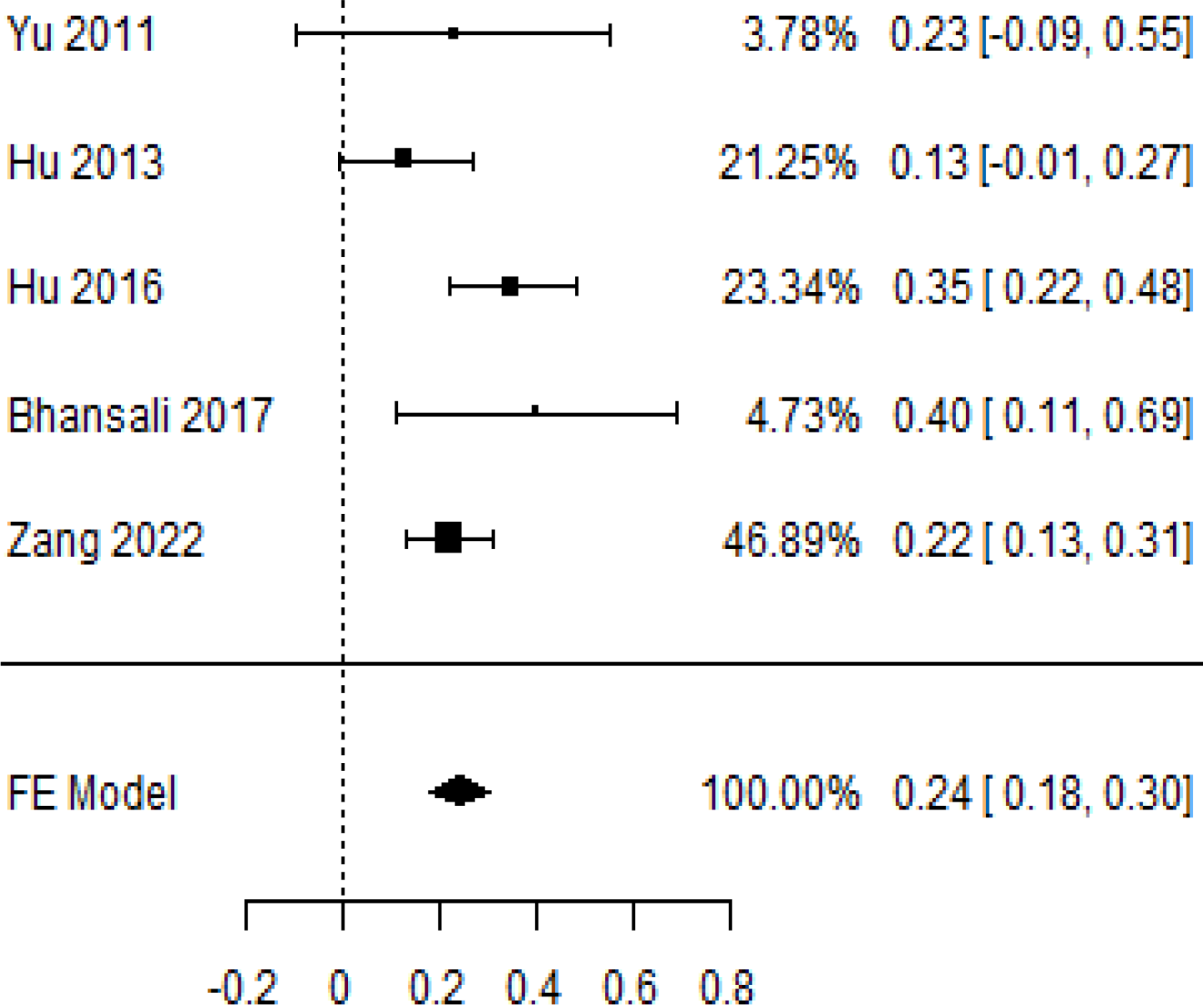	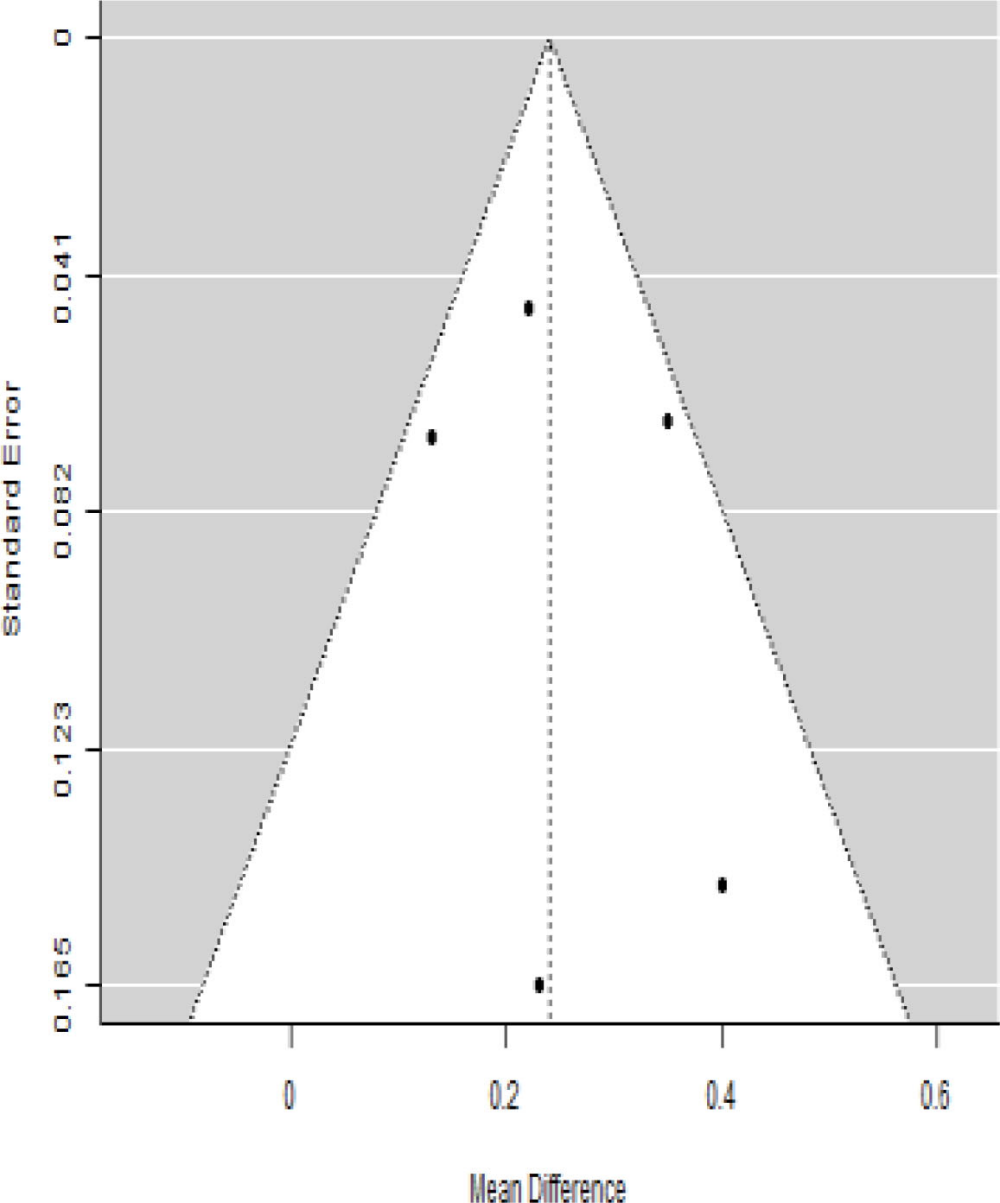
$Tau^{2}$ ( $\tau^{2}$ )	0.000		
$I^{2}$	39.81%		
d.f	4.000		
Q-test ( $\chi^{2}$ )	6.646		
P−value	0.156		
Test for overall effect			
Z−test	7.51		
P−value	< 0.001		
Publication bias assessment			
Begg and Mazumdar ( P−value )	1.000		
Egger’s regression ( P−value )	0.465		
12-month follow-up Heterogeneity		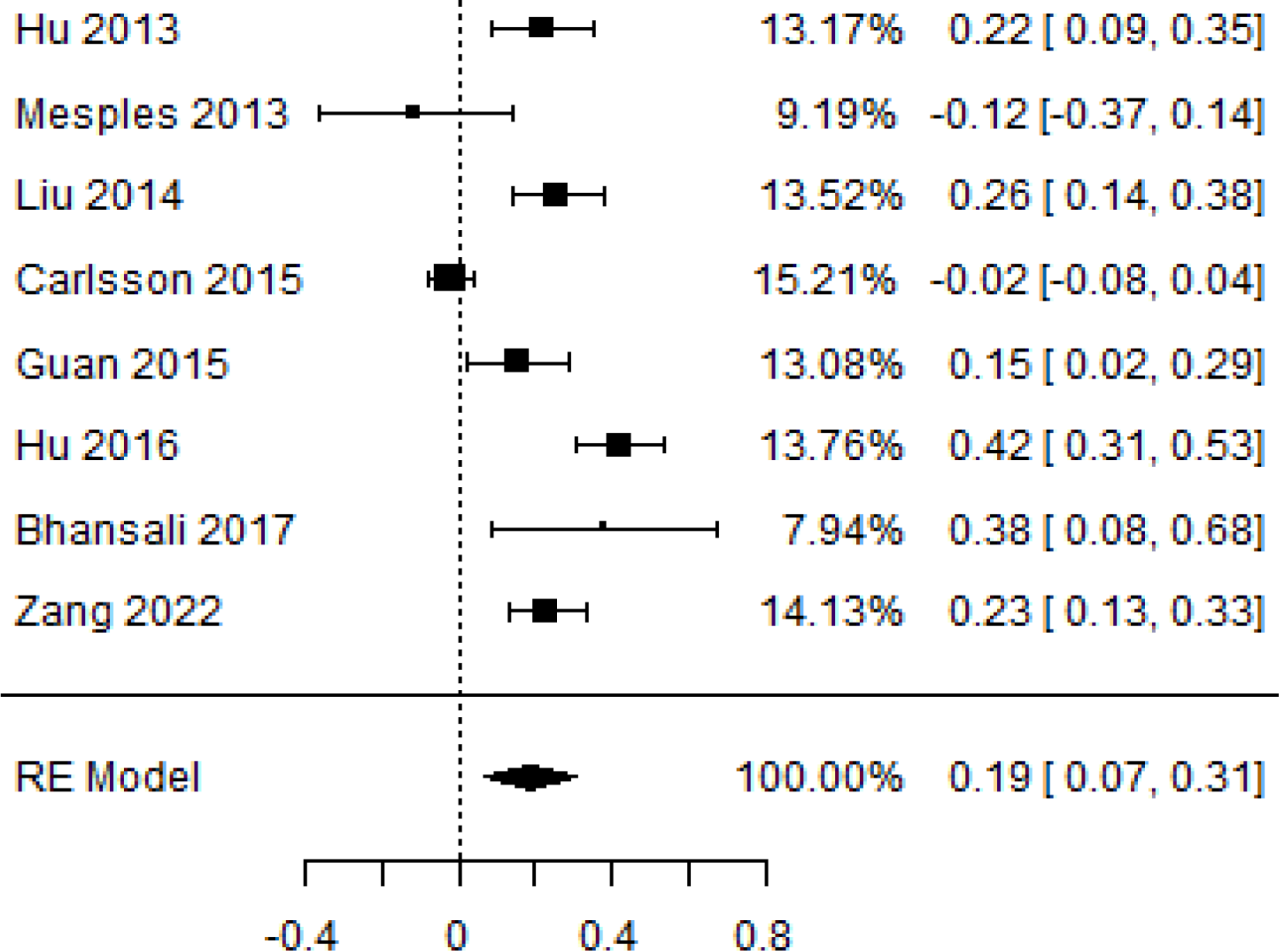	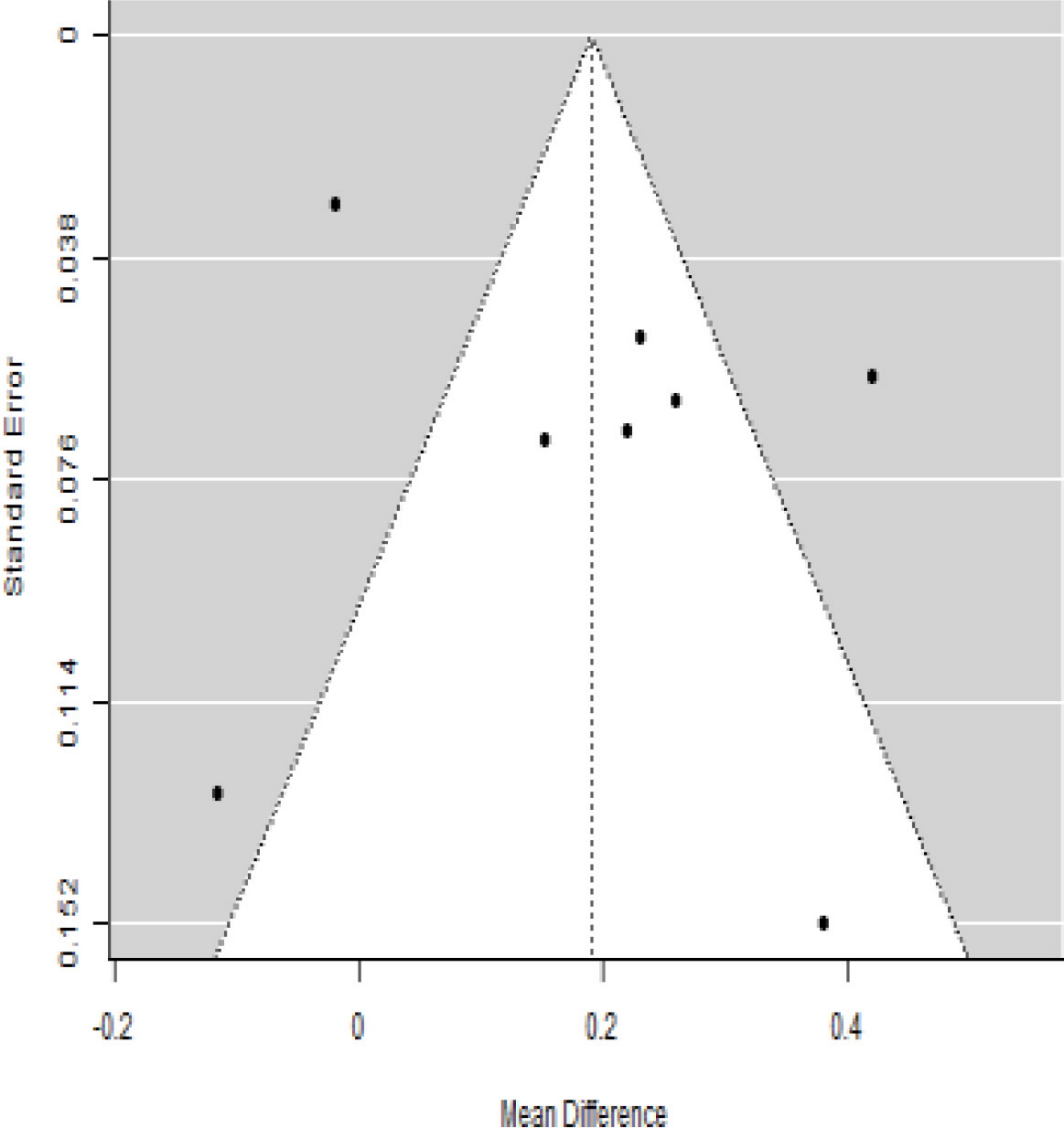
$Tau^{2}$ ( $\tau^{2}$ )	0.0233		
$I^{2}$	86.85%		
d.f	7.000		
Q-test ( $\chi^{2}$ )	68.604		
P−value	< 0.001		
Test for overall effect			
Z−test	3.15		
P−value	0.002		
Publication bias assessment			
Begg and Mazumdar ( P−value )	0.720		
Egger’s regression ( P−value )	0.892		

**Table 4:** Forest plots with the corresponding 95% CIs for the mean difference (MD) of fasting blood glucose.

	Statistics	Forest Plot Studies MD [95% CI] Weight (%) MD [95% CI]	Funnel Plot
3 Months Follow up Heterogeneity		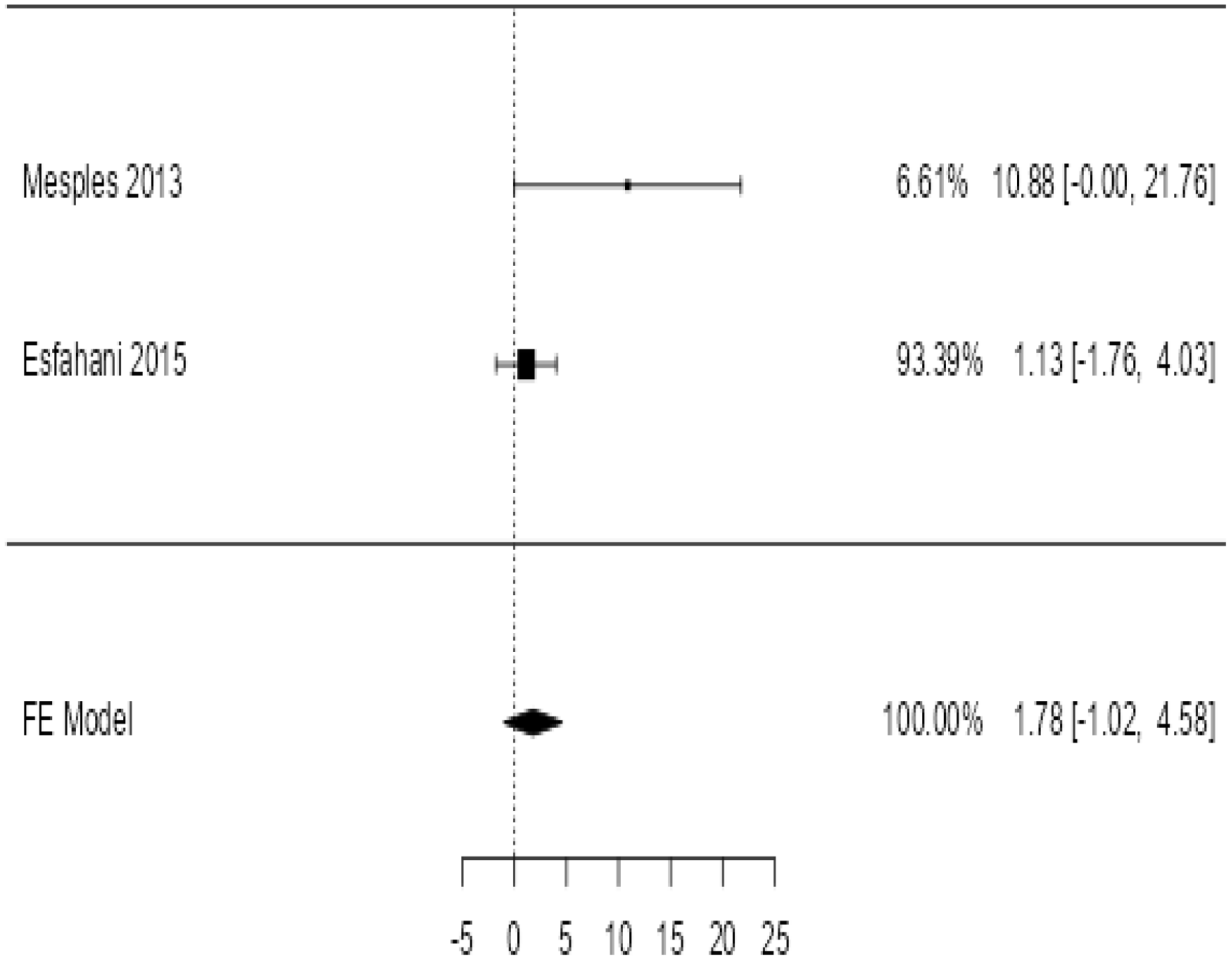	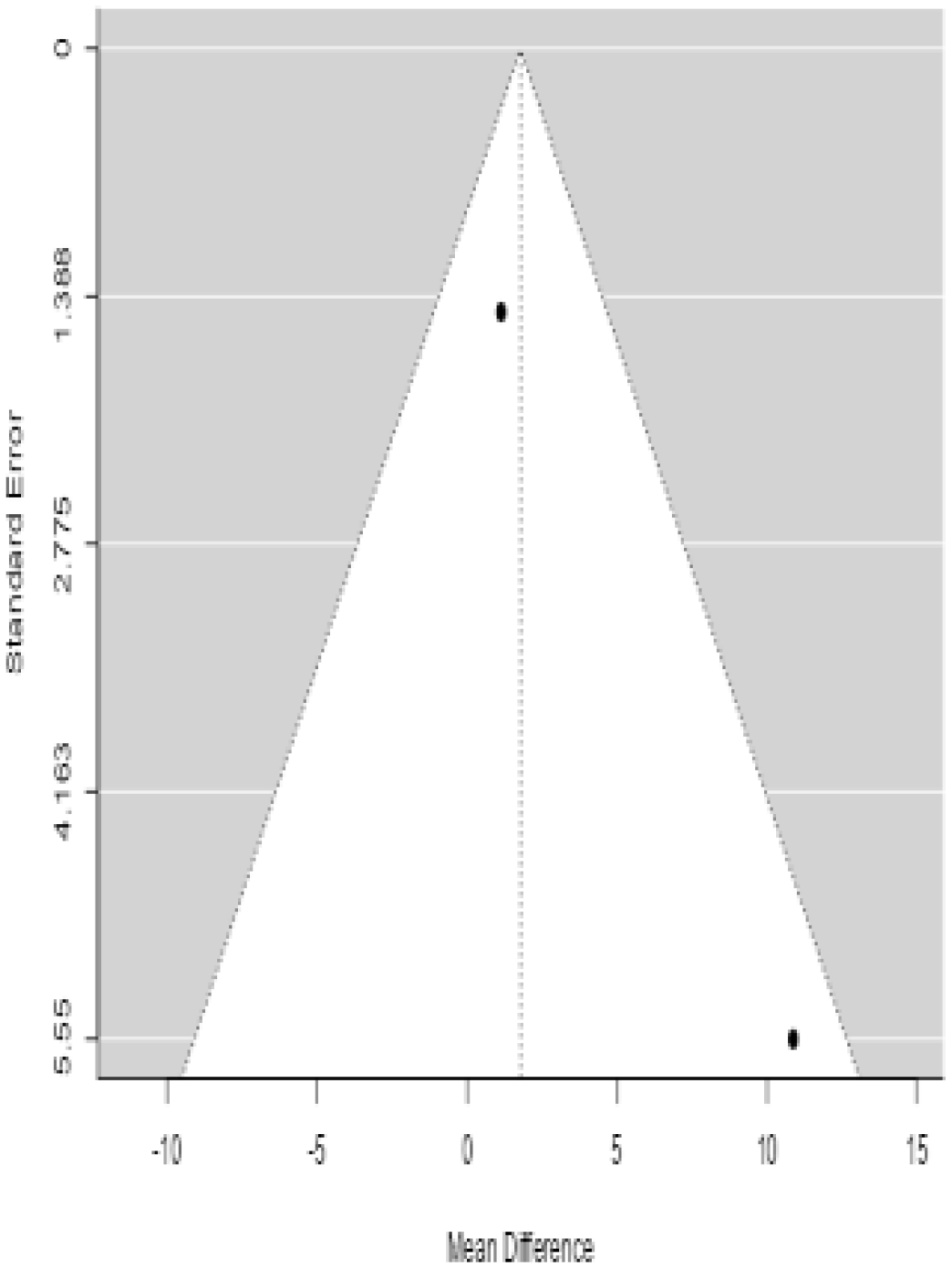
*Tau*^2^ (*τ*^2^)	0.000		
*I*^2^	62.25%		
*d.f*	1.000		
Q-test (*χ*^2^)	2.878		
*P - value*	0.090		
Test for overall effect			
*Z – test*	1.25		
*P - value*	0.212		
Publication Bias Assessment			
Begg & Mazumdar (*P - value*)	1.000		
Egger's Regression (*P - value*)	0.316		

**Table 5:** Forest plots with the corresponding 95% CIs for the mean difference (MD) of fasting plasma glucose.

	Statistics	Forest plot Studies MD [95% CI] Weight (%) MD [95% CI]	Funnel plot
3-month follow-up Heterogeneity		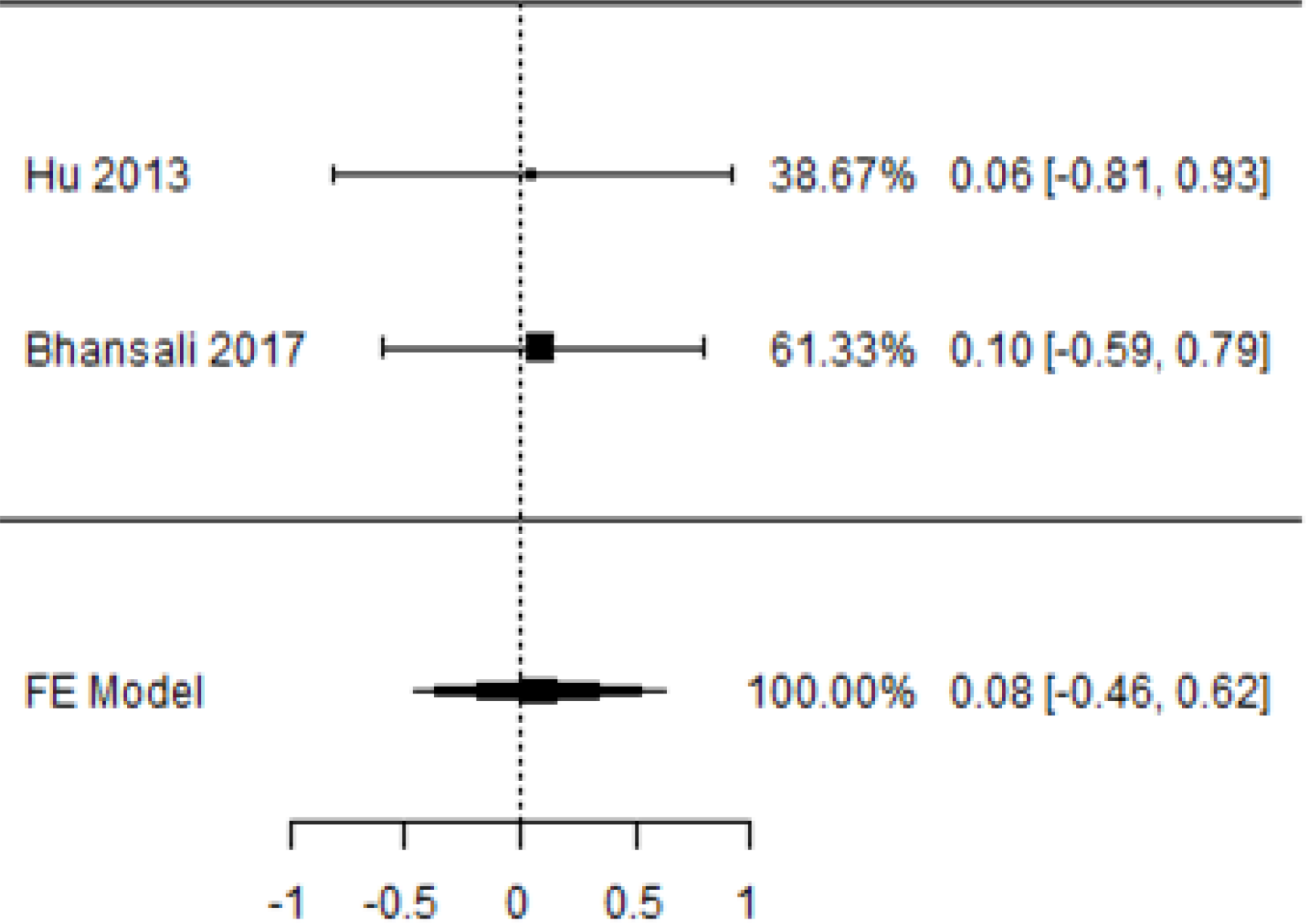	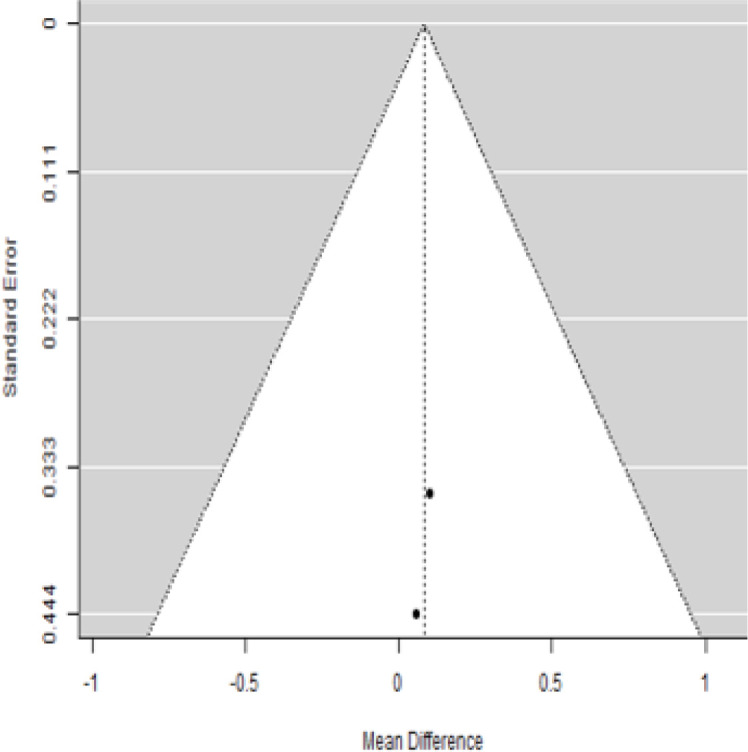
$Tau^{2}$ ( $\tau^{2}$ )	0.000		
$I^{2}$	0%		
d.f	1.000		
Q-test ( $\chi^{2}$ )	0.006		
P−value	0.937		
Test for overall effect			
Z−test	0.300		
P−value	0.764		
Publication bias assessment			
Begg and Mazumdar ( P−value )	1.000		
Egger’s regression ( P−value )	0.936		
6-month follow-up Heterogeneity		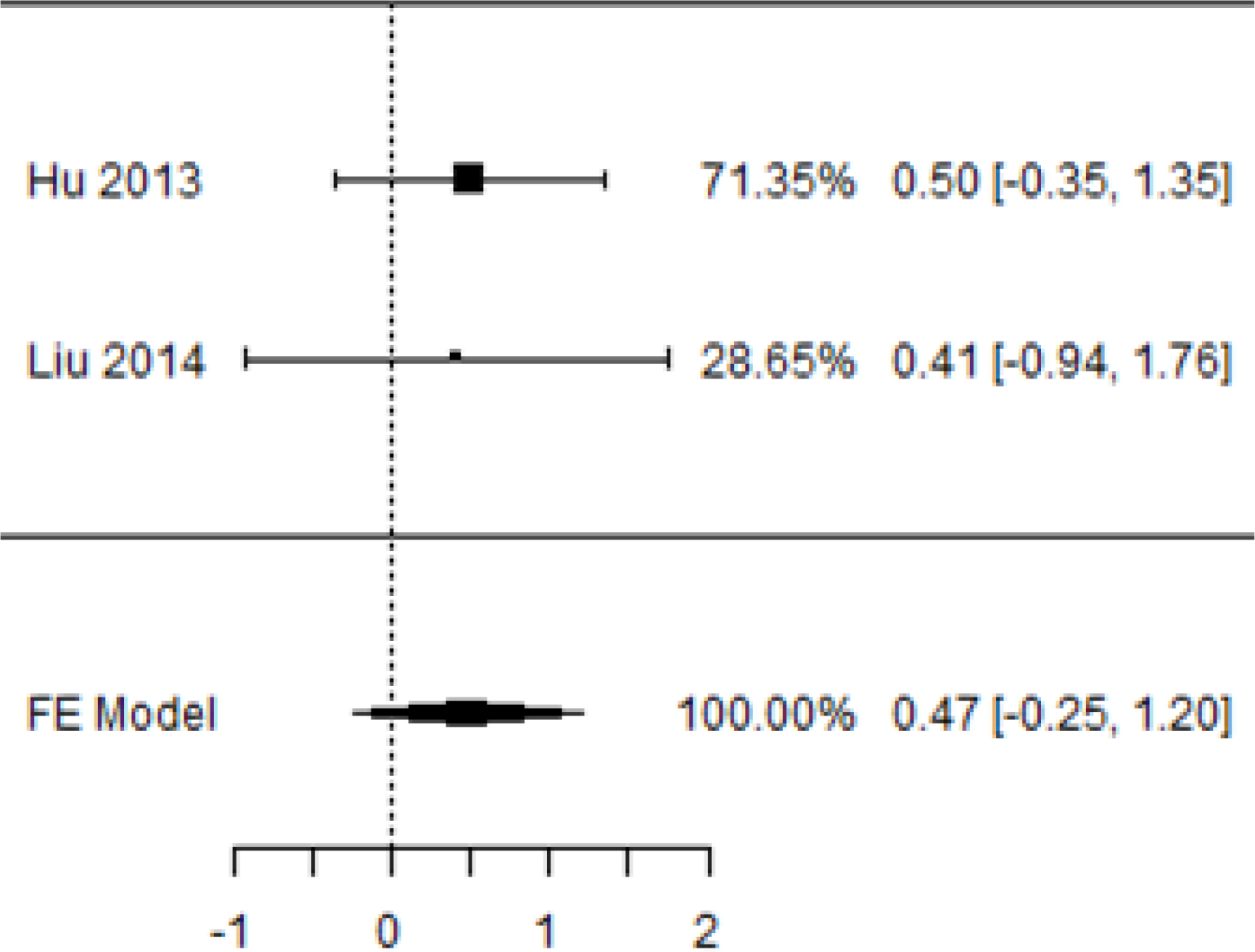	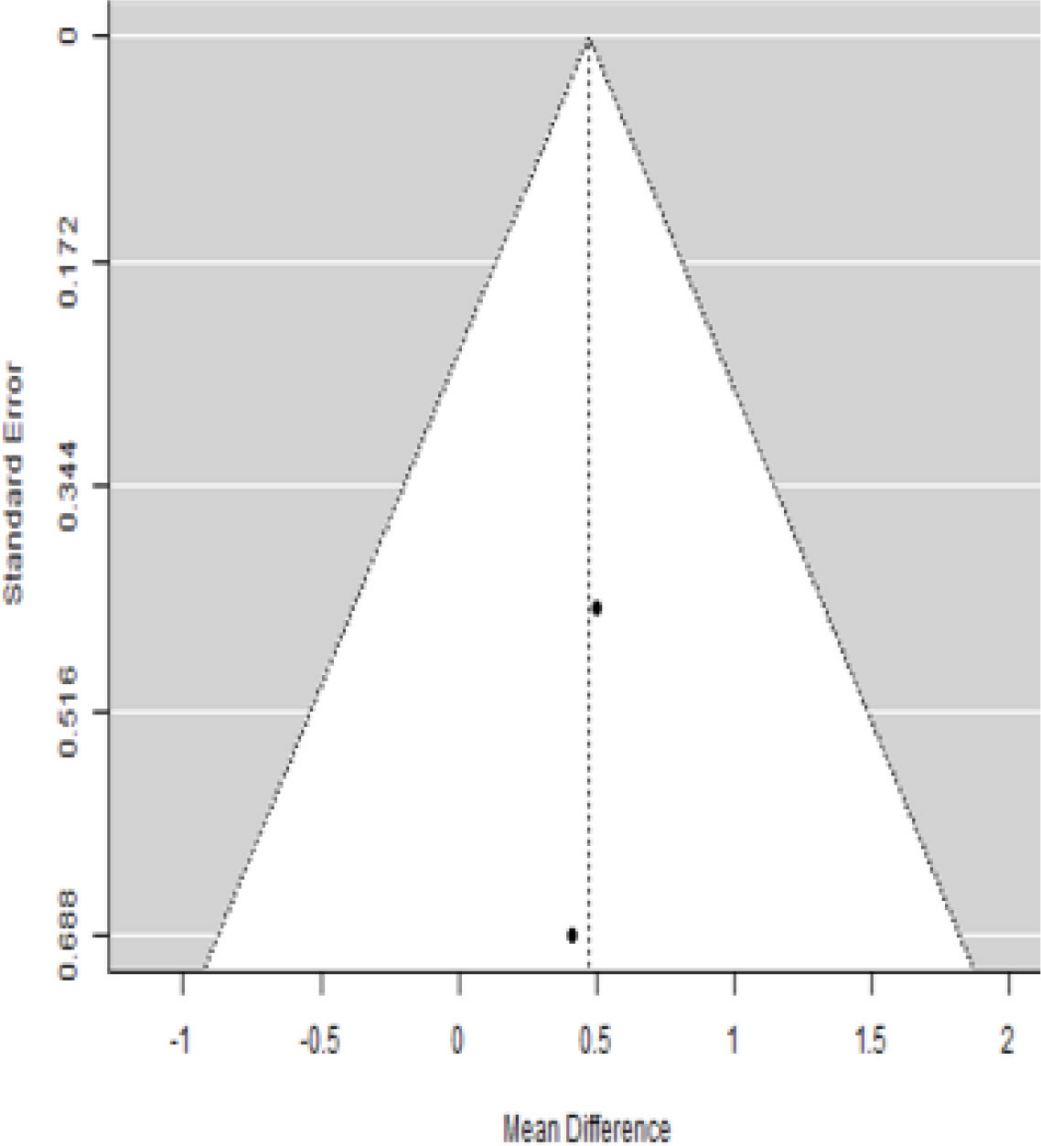
$Tau^{2}$ ( $\tau^{2}$ )	0		
$I^{2}$	0%		
d.f	1.000		
Q-test ( $\chi^{2}$ )	0.012		
P−value	0.912		
Test for overall effect			
Z−test	1.29		
P−value	0.198		
Publication bias assessment			
Begg and Mazumdar ( P−value )	0.109		
Egger’s regression ( P−value )	0.912		
9-month follow-up Heterogeneity		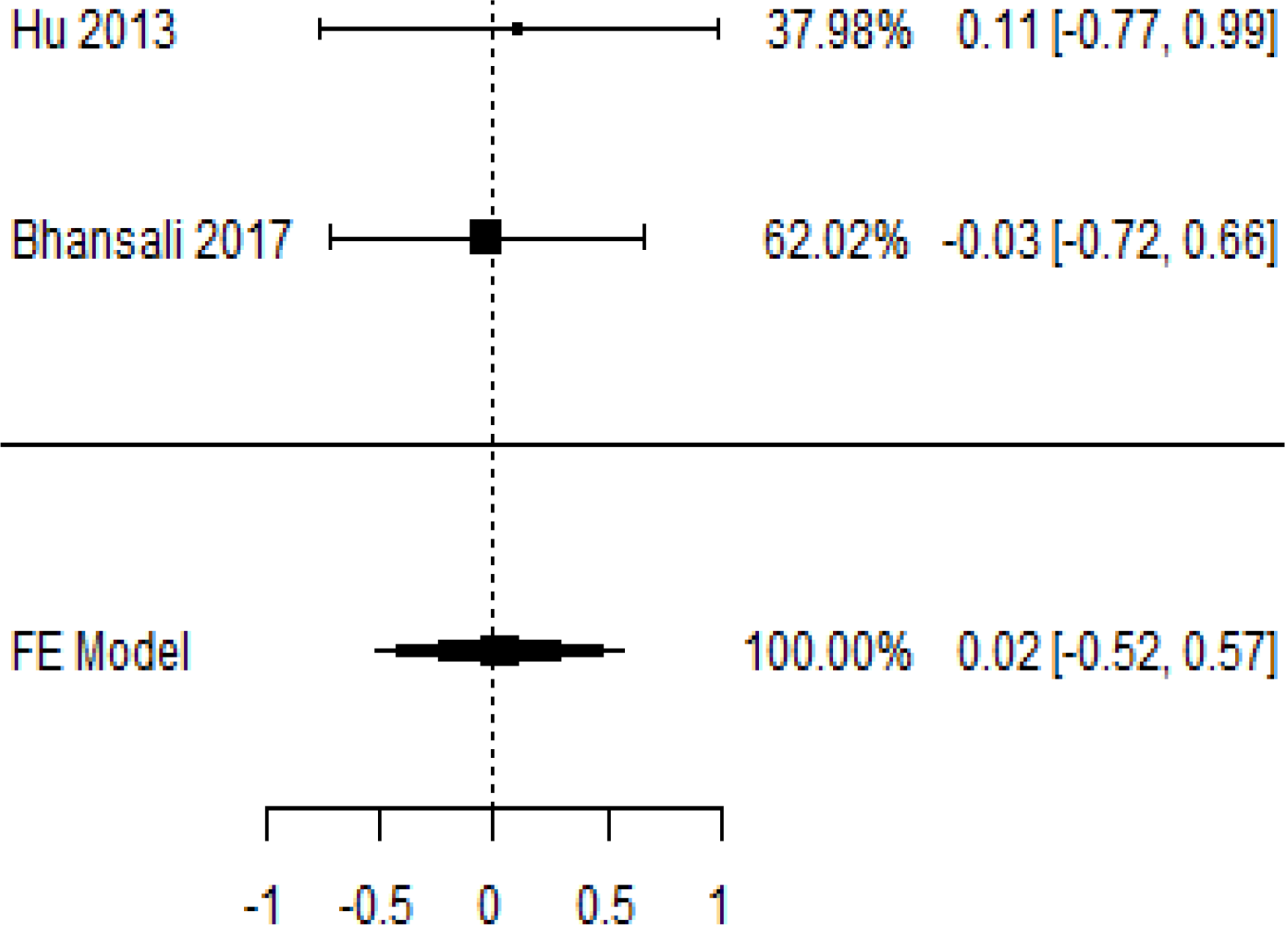	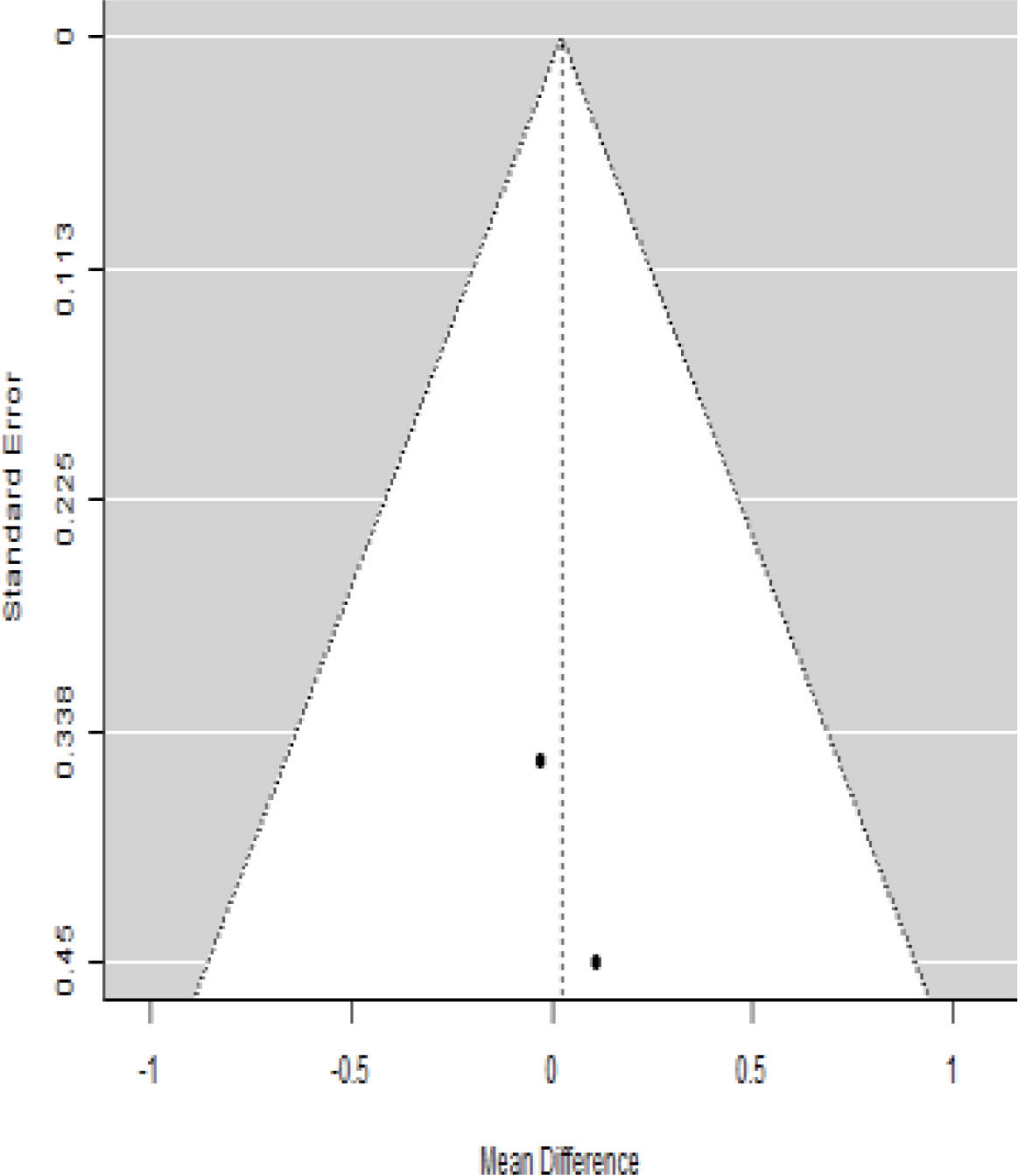
$Tau^{2}$ ( $\tau^{2}$ )	0		
$I^{2}$	0%		
d.f	1.000		
Q-test ( $\chi^{2}$ )	0.061		
P−value	0.805		
Test for overall effect			
Z−test	0.0849		
P−value	0.932		
Publication bias assessment			
Begg and Mazumdar ( P−value )	1.000		
Egger’s regression ( P−value )	0.805		
12-month follow-up Heterogeneity		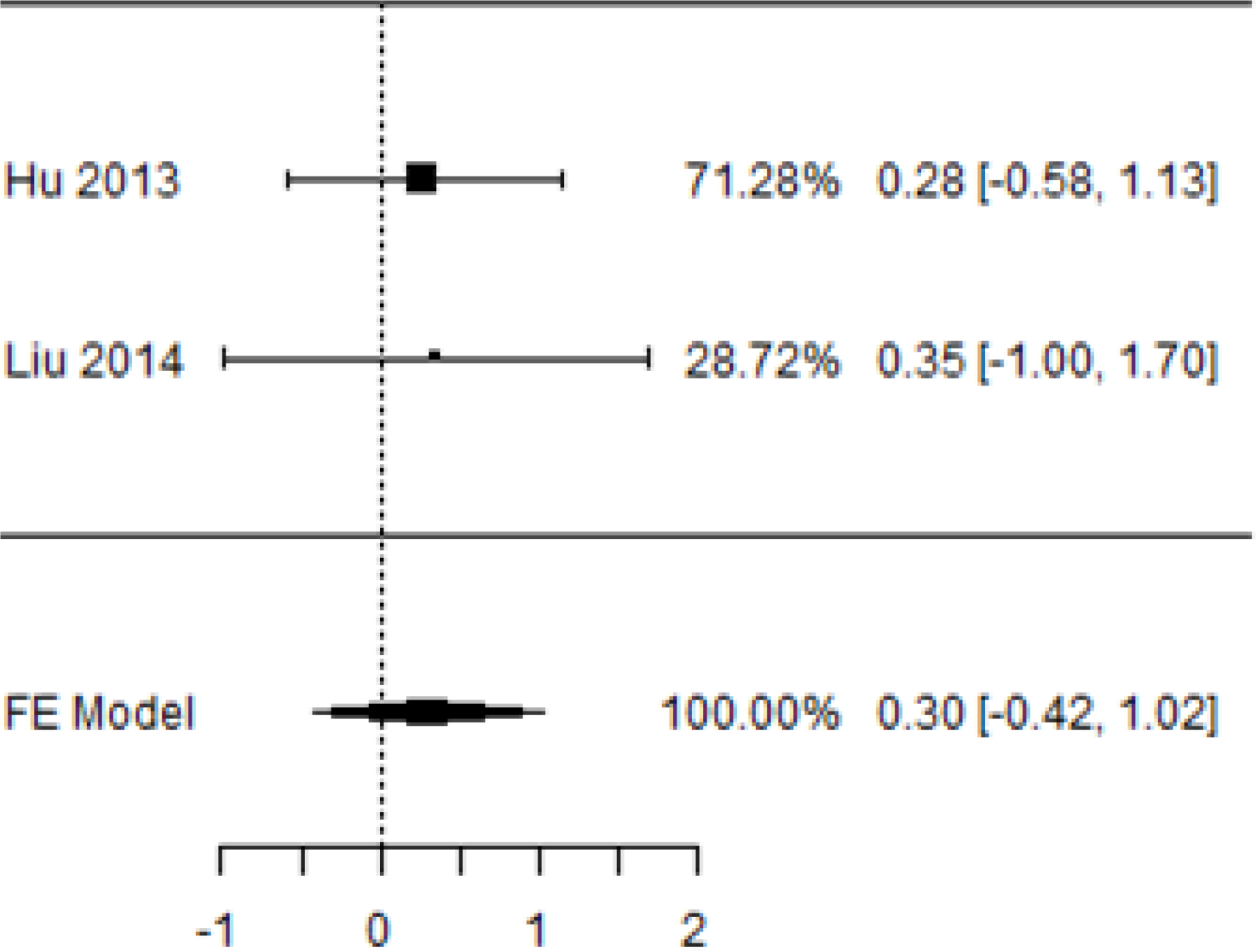	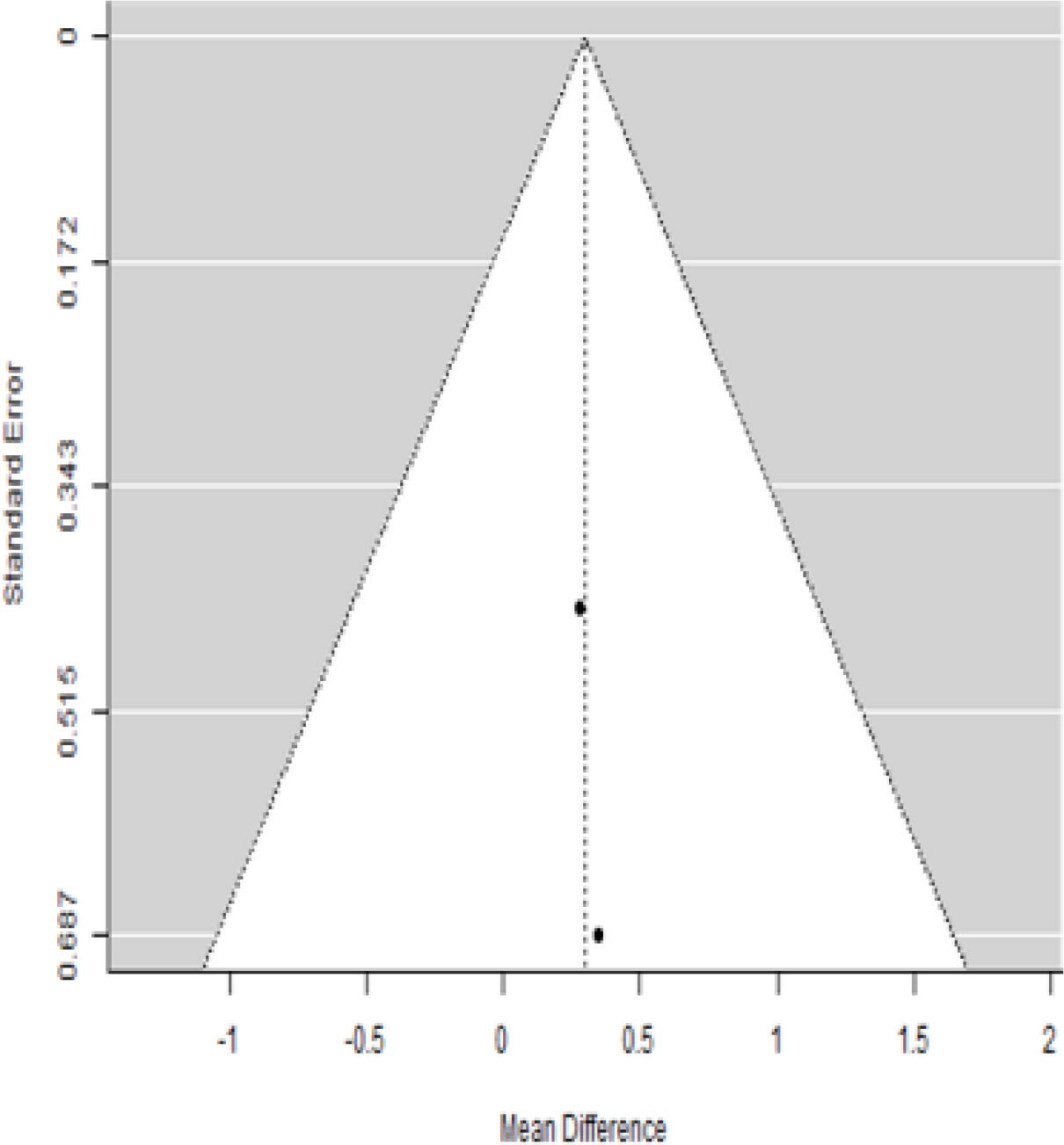
$Tau^{2}$ ( $\tau^{2}$ )	0		
$I^{2}$	0%		
d.f	1.000		
Q-test ( $\chi^{2}$ )	0.008		
P−value	0.929		
Test for overall effect			

**Table 6:** Forest plots with the corresponding 95% CIs for the mean difference (MD) of C-peptide.

	Statistics	Forest plot Studies MD [95% CI] Weight (%) MD [95% CI]	Funnel plot
3-month follow-up Heterogeneity		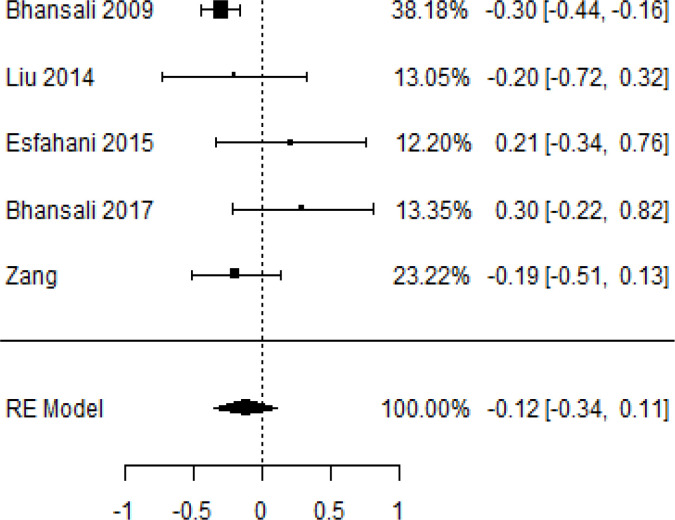	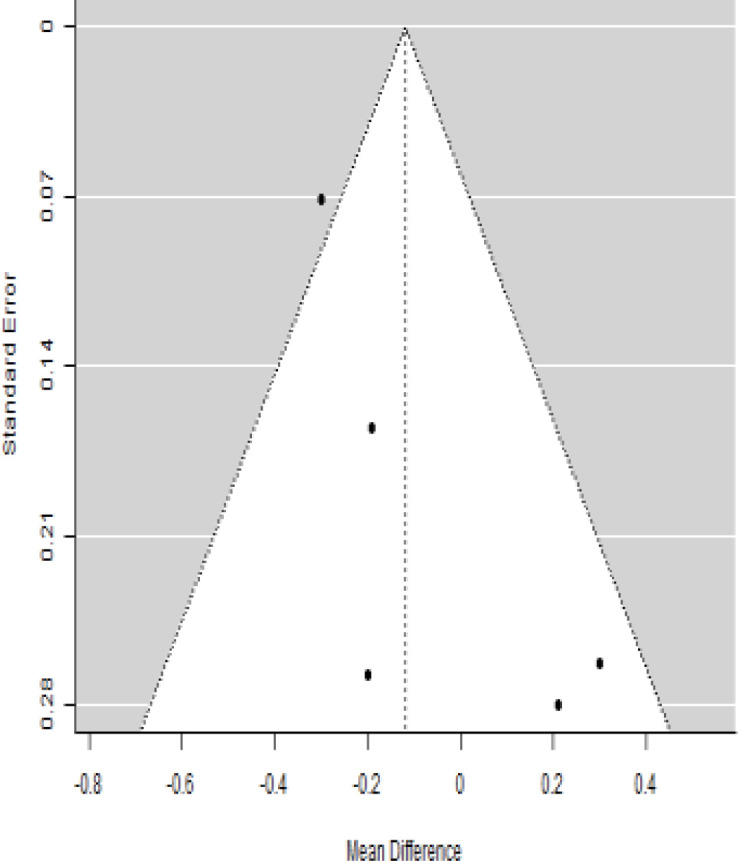
$Tau^{2}$ ( $\tau^{2}$ )	0.0295		
$I^{2}$	48.39%		
d.f	4.000		
Q-test ( $\chi^{2}$ )	7.570		
P−value	0.109		
Test for overall effect			
Z−test	-1.04		
P−value	0.300		
Publication bias assessment			
Begg and Mazumdar ( P−value )	0.483		
Egger’s regression ( P−value )	0.020		
6-month follow-up Heterogeneity		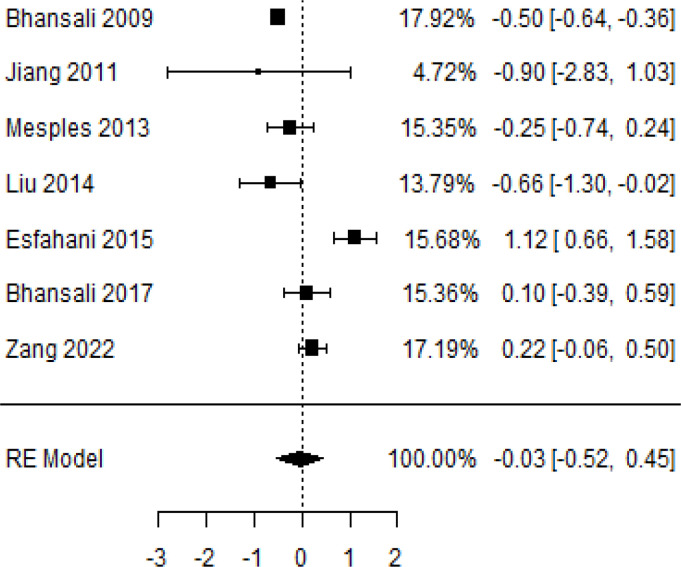	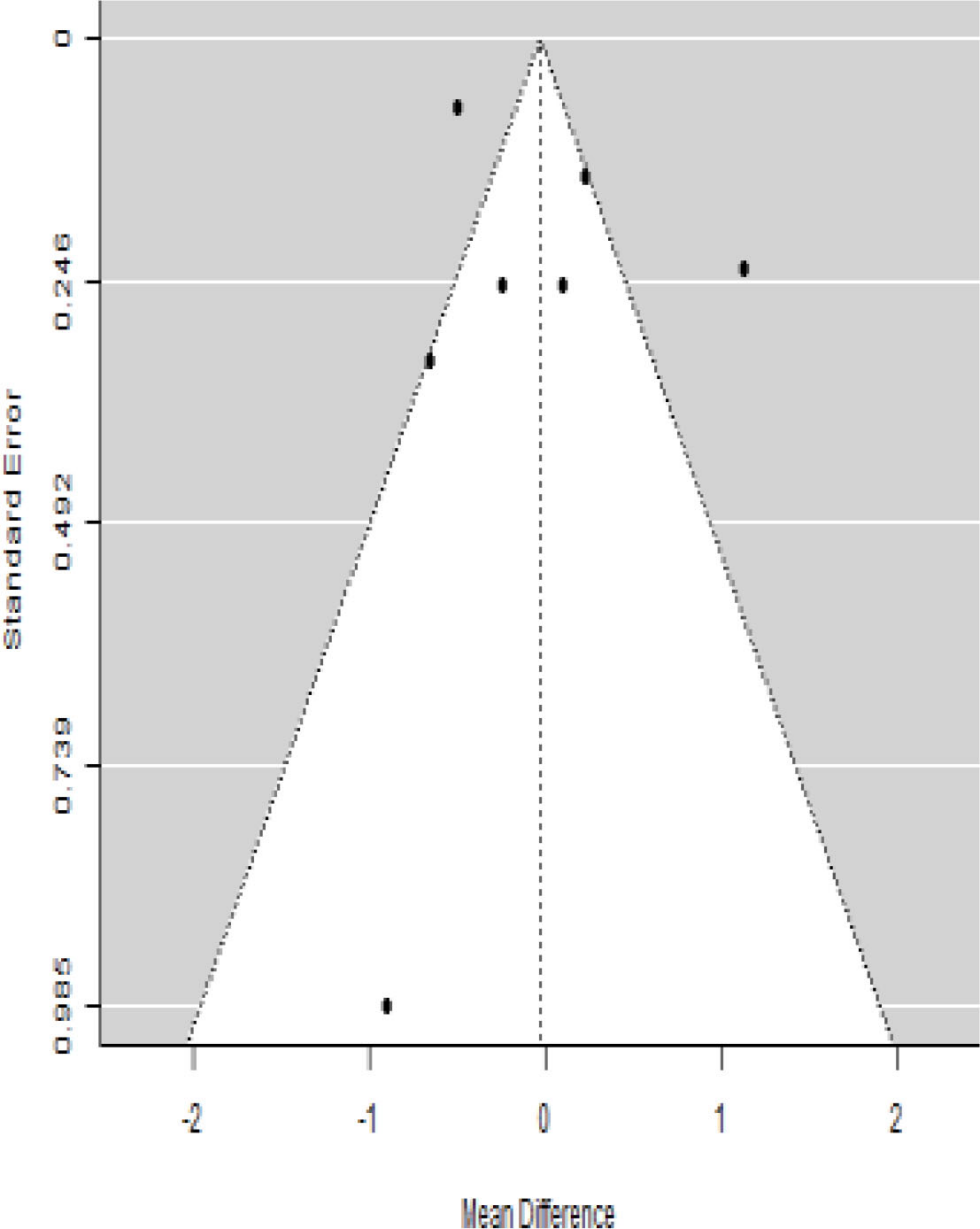
$Tau^{2}$ ( $\tau^{2}$ )	0.3398		
$I^{2}$	90.66%		
d.f	6.000		
Q-test ( $\chi^{2}$ )	62.159		
P−value	< 0.001		
Test for overall effect			
Z−test	-0.132		
P−value	0.895		
Publication bias assessment			
Begg and Mazumdar ( P−value )	0.562		
Egger’s regression ( P−value )	0.517		
12-month follow-up Heterogeneity		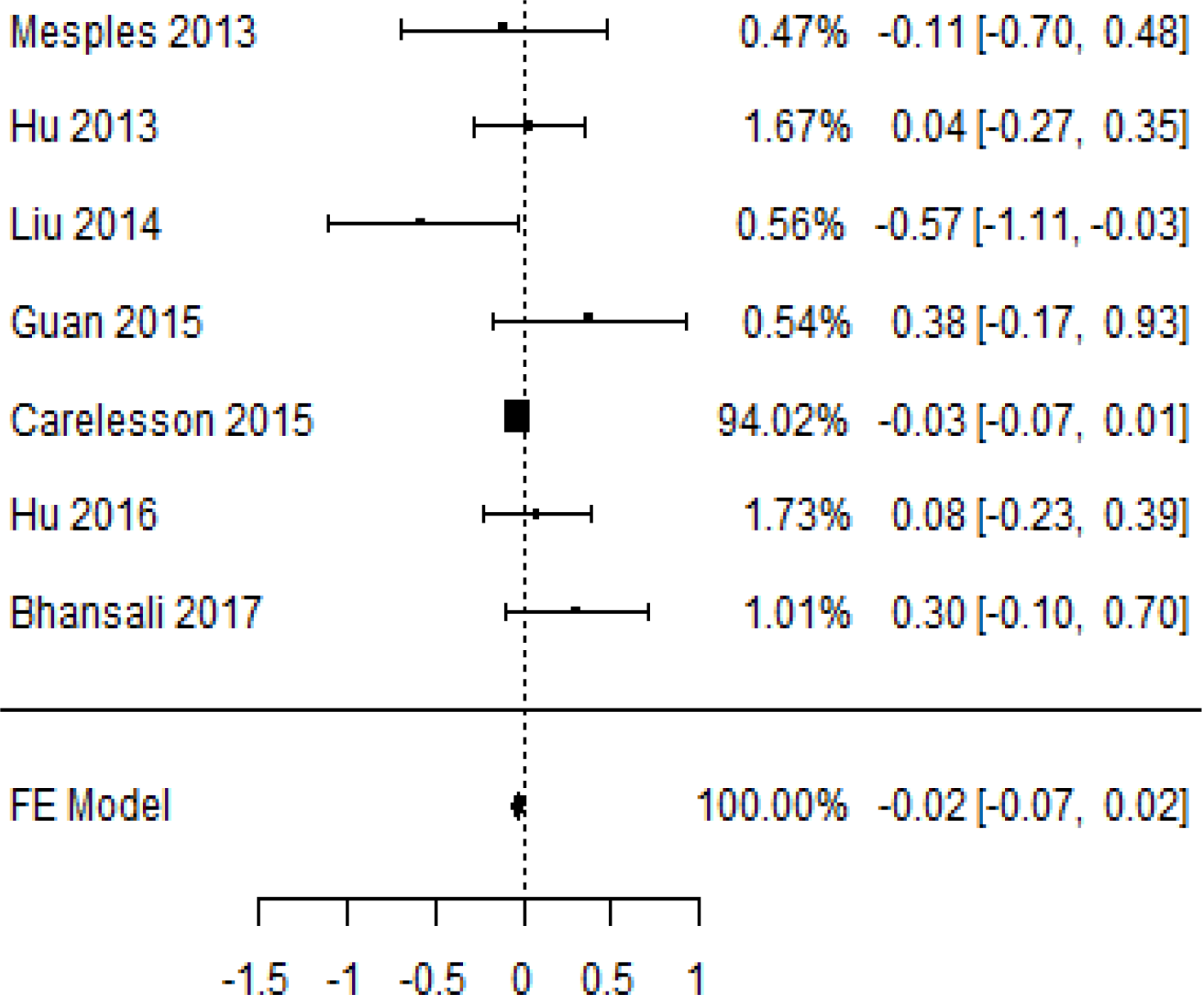	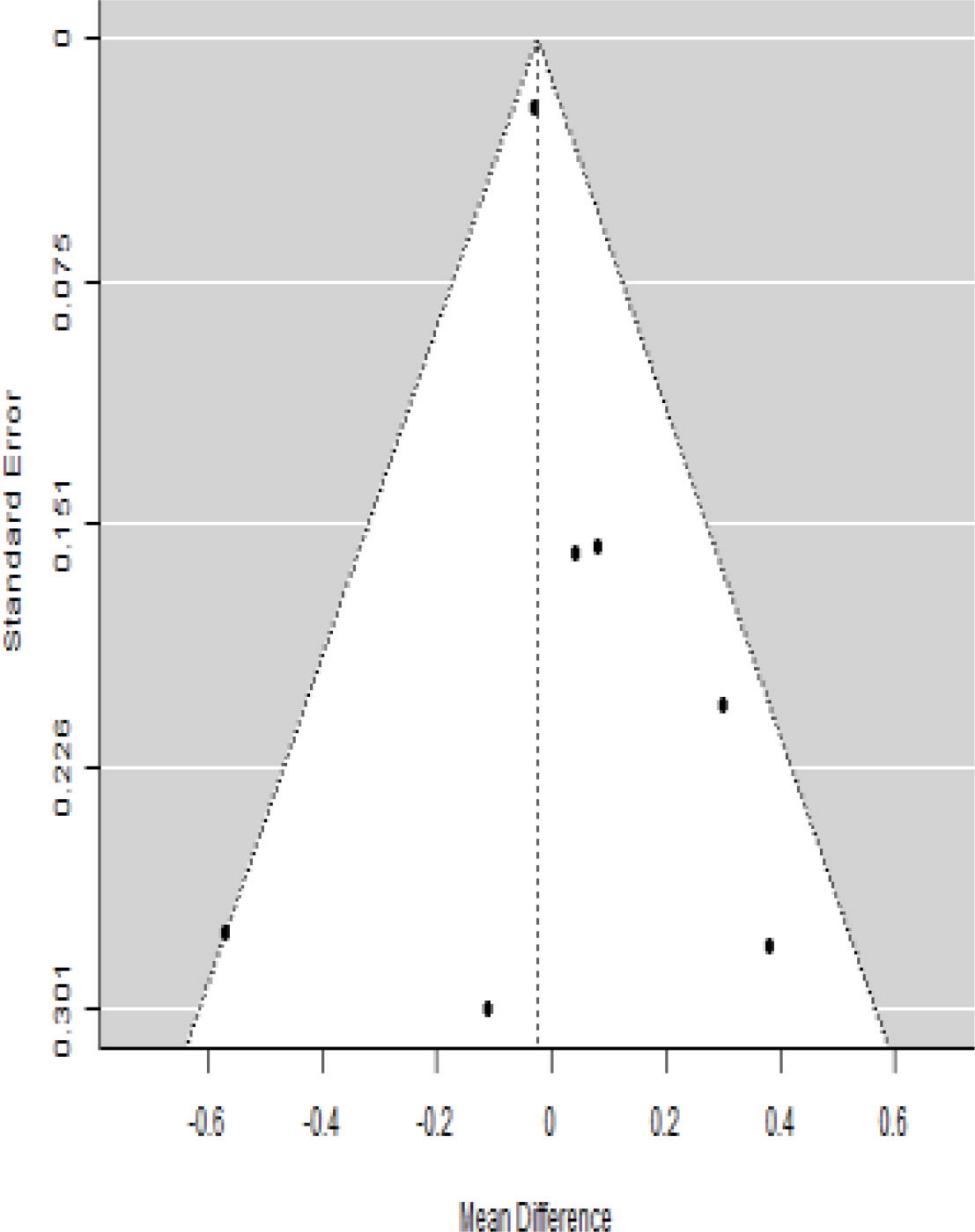
$Tau^{2}$ ( $\tau^{2}$ )	0.000		
$I^{2}$	34.51%		
d.f	6.000		
Q-test ( $\chi^{2}$ )	9.162		
P−value	0.165		
Test for overall effect			
Z−test	-1.20		
P−value	0.231		
Publication bias assessment			
Begg and Mazumdar ( P−value )	1.000		
Egger’s regression ( P−value )	0.460		

## Results

### Search results

**Figure 1:**
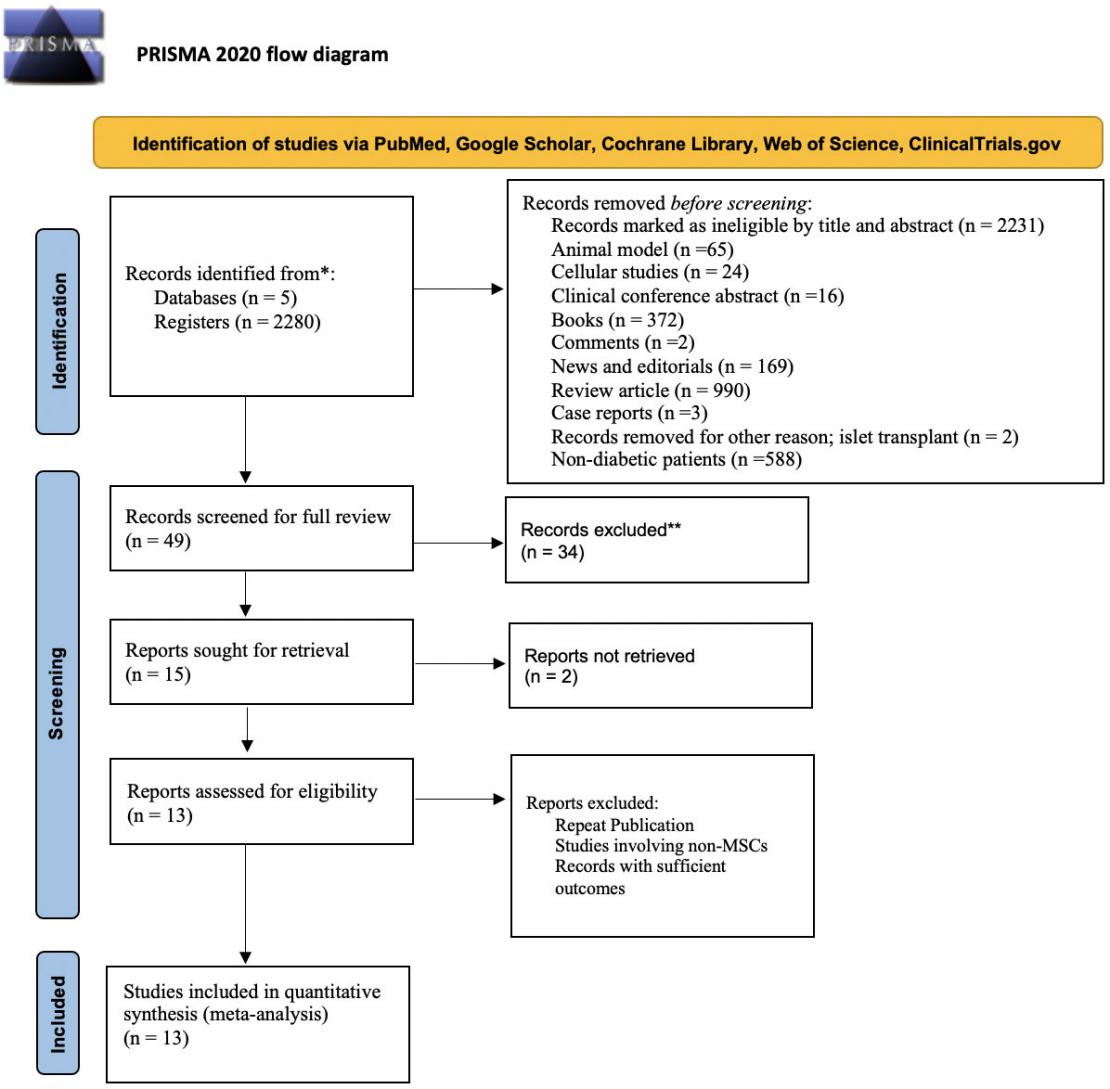
Flow diagram illustrating the identification, screening, and selection of the eligible clinical trials/studies for meta-analysis.

The search strategy identified a total of 2,280 articles from selected databases and prior bibliographies. Following a review of the titles and abstracts, 2,231 studies were eliminated due to their lack of relevance in terms of purpose, goal, intervention, and/or measures. After a thorough evaluation of the remaining 49 papers, 34 were excluded. In total, 15 clinical studies met the inclusion criteria and were embraced in the quantitative data analysis for selected outcome measures. However, two of the studies (41, 42) were unable to be retrieved. We searched them on different resources, but they were inaccessible. Finally, 13 clinical studies (15–17, 28–38), consisting of 302 subjects, were embraced in the meta-analysis. The selection process of studies is often presented in a flow diagram, which may be visualized in **Figure 1**. This diagram provides an overview of the steps taken to identify, screen, and include studies in a systematic review or meta-analysis.

### Attributes of the included studies

**Table 1** presents clinical data from studies that were included in the analysis. The studies were published between 2000 and November 2023 and included sample sizes ranging from 1 to 73. The subjects’ mean age varied from 17.6 to 57.6 years, with a predominance of male participants. Some trials, however, were unable to collect enough clinical data, such as body mass, blood pressure, liver and renal function tests, and fasting plasma insulin. The eligible studies involved four types of MSCs: including BM-MSCs, Wharton’s jelly-derived (WJ-MSCs), and UC-MSCs, with various cell doses used. The intervention regimen involved administering MSCs via intravenous or intra-arterial delivery, with doses ranging from (0.88 ± 0.05) × 10^6^ to (1.2 ± 0.3) × 10^9^. The follow-up period varied from =3 to 12 months. Of the eligible studies, six reported data on MSCs and T1DM (28–31, 33–35), and seven reported data on MSCs and T2DM (15–17, 32, 36–38).

### Effects of stem cell transplantation on HbA1c (%)

In **Table 2**, the results of the meta-analysis for the parameter HbA1c with 3-, 6-, 9-, and 12-month follow-up demonstrated that the MSC transplantation is associated with a reduction of HbA1c. It was also found that the MSCs had a significant effect on both T1DM and T2DM with 3-, 6-, 9-, and 12-month follow-up. According to the forest plots, the overall effect size measured with mean difference (MD) revealed comparing the administration of the MSCs and baseline which had shown the significant reduction in HbA1c at 5% level of significance in 3-, 6-, 9-, and 12-month follow-up as (MD: 0.68, 95% CI: 0.29 to 1.07, *P*-value:< 0.001, I^2^: 58.33%), (MD: 0.18, 95% CI: 0.07 to 0.29, *P*-value:< 0.001, I2:84.84%), (MD: 1.89, 95% CI: -0.18 to 3.97, *P*-value: 0.074 > 0.05, I2: 96.73%), and (MD: 0.95, 95% CI: 0.33 to 1.57, *P*-value: 0.003< 0.05, I2: 87.33%), respectively.

**Figure 2:**
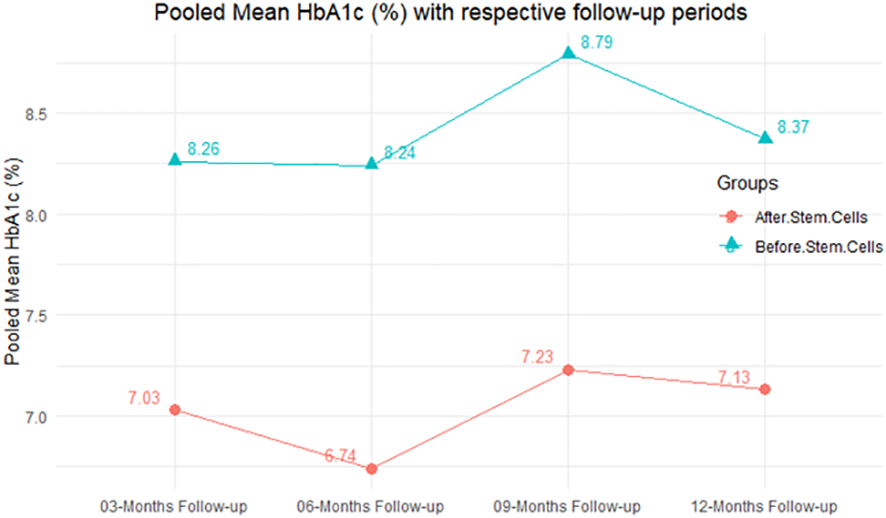
Pooled mean HbA1c (%) with respective follow-up periods.

In RCT, we observed that the HbA1c level was lower in the MSC-treated group than in the control group after 3, 6, and 12 months. Furthermore, the difference was statistically significant with 3-, 6-, and 12-month follow-up as (MD = 0.32, 95% CI 0.03 to 0.61, *P*-value = 0.028), (MD = 0.17, 95% CI 0.01 to 0.34, *P*-value = 0.043), and (MD = 0.95, 95% CI 0.12 to 1.77, *P*-value = 0.025), respectively, while in n-RCT, the HbA1c in the MSC-treated group showed a significant decrease from its baseline level to those at the 3- and 6-month follow-up period (MD = 0.96, 95% CI 0.70 to 1.22, *P*-value< 0.001), and (MD = 0.19, 95% CI 0.04 to 0.34, *P*-value = 0.012), respectively. The observed MDs in all included studies (100%) along with 3-, 6-, 9-, and 12-month follow-up were being positive (100%), which had indicated a decrease in HbA1c due to MSC transplantation (**Table 7**). Moreover, from graph/**Figure 2**, the pooled mean of HbA1c computed from all studies with 3-, 6-, 9-, and 12-month follow-up showed a reduction in the levels of HbA1c due to stem cell transplantation when compared with the control group.

**Table 7:** Summarized results about HbA1c, insulin requirement, fasting blood glucose, fasting plasma glucose, and C-peptide for n-RCT and RCT.

Parameters	Follow-up	n-RCT	RCT
HbA1c	3	(MD = 0.96, 95% CI 0.70 to 1.22, *P*-value*<* 0.001)	(MD = 0.32, 95% CI 0.03 to 0.61, *P*-value = 0.028)
	6	(MD = 0.19, 95% CI 0.04 to 0.34, *P*-value *=* 0.012)	(MD = 0.17, 95% CI 0.01 to 0.34, *P*-value = 0.043)
	12	–	(MD = 0.95, 95% CI 0.12 to 1.77, *P*-value = 0.025)
Insulin requirement	3	(MD = 0.19, 95% CI 0.04 to 0.34, *P*-value *=* 0.012)	(MD = 0.17, 95% CI 0.01 to 0.34, *P*-value *=* 0.043)
	6	(MD = 0.08, 95% CI -0.46 to 0.61, *P*-value = 0.282)	(MD = 0.22, 95% CI 0.10 to 0.35, *P*-value*<* 0.001)
	9	–	(MD = 0.24, 95% CI 0.18 to 0.30, *P*-value*<* 0.001)
	12	(MD = 0.21, 95% CI 0.12 to 0.30, *P*-value*<* 0.001)	(MD = 0.19, 95% CI 0.02 to 0.35, *P*-value = 0.021)
FPG	3	–	(MD = 0.74, 95% CI -0.54 to 2.02, *P*-value = 0.258)
	6	(MD = 0.93, 95% CI 0.14 to 1.72, *P*-value = 0.021)	(MD = 0.20, 95% CI -0.34 to 0.73, *P*-value = 0.471)
	9	–	(MD = 0.02, 95% CI -0.52 to 0.57, *P*-value*<* = 0.932)
	12	–	(MD = -1.11, 95% CI -3.10 to 0.88, *P*-value = 0.273)
C-peptide	3	(MD = -0.27, 95% CI -0.40 to -0.14, *P*-value*<* 0.001)	(MD = -0.05, 95% CI -0.33 to 0.22, *P*-value = 0.712)
	6	(MD = -0.01, 95% CI -1.12 to 1.11, *P*-value = 0.991)	(MD = 0.06, 95% CI -0.22 to 0.33, *P*-value *=* 0.690)
	12	–	(MD = -0.02, 95% CI -0.06 to 0.02, *P*-value *=* 0.250)

### Effects of stem cell transplantation on insulin (IU/kg/day) requirement

According to the forest plots presented in **Table 3**, the overall effect size evaluated using difference (MD) demonstrated a substantial decrease in insulin requirement between the administration of MSCs and the control group at 5% level of significance in 3-, 6-, 9-, and 12-month follow-up as (MD: 0.18, 95% CI: 0.07 to 0.29, *P*-value:< 0.001, *I*^2^: 84.84%), (MD: 0.17, 95% CI: -0.04 to 0.38, *P*-value: 0.112 > 0.05, *I*^2^: 88.49%), (MD: 0.24, 95% CI: 0.18 to 0.30, *P*-value:< 0.001, *I*^2^: 39.81%), and (MD: 0.19, 95% CI: 0.07 to 0.31, *P*-value: 0.002< 0.05, *I*^2^: 86.85%), respectively.

**Figure 3:**
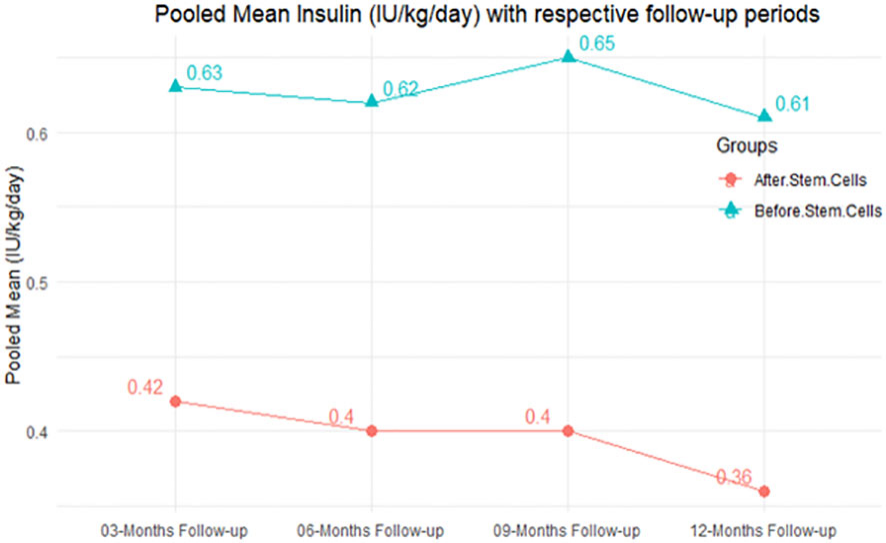
Pooled mean insulin requirement (IU/kg/day) with respective follow-up periods.

In RCT, it was shown that the insulin requirement level was lower in the MSC-treated group than in the control group after 3, 6, 9, and 12 months of follow-up. Moreover, the difference was statistically significant with 3-, 6-, 9-, and 12-month follow-ups as (MD = 0.17, 95% CI 0.01 to 0.34, *P*-value = 0.043), (MD = 0.22, 95% CI 0.10 to 0.35, *P*-value< 0.001), (MD = 0.24, 95% CI 0.18 to 0.30, *P*-value< 0.001), and (MD = 0.19, 95% CI 0.02 to 0.35, *P*-value = 0.021), respectively, while in n-RCT the insulin requirement in the MSC-treated group showed a significant decrease from its baseline level to those at the 3-, 6-, and 12-month follow-up period (MD = 0.19, 95% CI 0.04 to 0.34, *P*-value = 0.012) and (MD = 0.21, 95% CI 0.12 to 0.30, *P*-value< 0.001), respectively. However, the difference was not statistically significant at 6 months (MD = 0.08, 95% CI -0.46 to 0.61, *P*-value = 0.282) (**Table 7**). The observed MDs in all included studies (100%) along with 3-, 6-, 9-, and 12-month follow-up were being positive (100%), which had indicated a reduction in insulin due to MSCs therapy. Furthermore, from graph/**Figure 3**, the pooled mean of insulin requirement computed from all studies with 3-, 6-, 9-, and 12-month follow-up showed a reduction in the levels of insulin requirement due to stem cell transplantation.

### Effects of stem cell transplantation on FBG (mmol/L)

**Figure 4:**
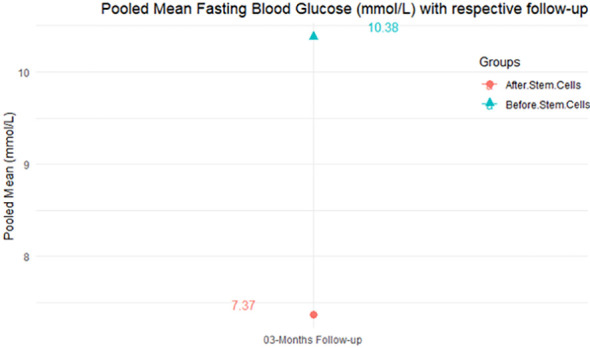
Pooled mean fasting blood glucose (mmol/L) with respective follow-up periods.

From **Table 4**, according to the forest plots, the overall effect size measured with difference (MD) showed that when the MSCs were given to the control group, the fasting blood glucose level dropped by a fair number at 5% level of significance in 6-month follow-up as (MD: 1.78, 95% CI: -1.02 to 4.58, *P*-value: 0.212, *I*^2^: 62.25%). The observed MDs in all included studies (100%) along with 6-month follow-up were being positive (100%), which indicated a decline in fasting blood glucose due to MSC transplantation. In addition, from graph/**Figure 4**, the pooled mean of FBG was computed from all studies with 3-month follow-up.

### Effects of stem cell transplantation on FPG (mmol/L)

In **Table 5**, the total effect size evaluated by mean difference (MD) revealed by comparing the MSC administration and the baseline group showed a considerably lower fasting plasma glucose, as represented by the forest plots at 5% level of significance in 3-, 6-, 9-, and 12-month follow-up as (MD: 0.08, 95% CI: -0.46 to 0.62, *P*-value: 0.764 > 0.05, *I*^2^: 0%), (MD: 0.47, 95% CI: -0.25 to 1.20, *P*-value: 0.198 > 0.05, *I*^2^: 0%), (MD: 0.02, 95% CI: -0.52 to 0.57, *P*-value: 0.061 > 0.05, *I*^2^: 0%), and (MD: 0.30, 95% CI: -0.42 to 1.02, *P*-value: 0.417 > 0.05, *I*^2^: 0%).

In RCT, we observed that the FPG level was lower in the MSC-treated group than in the control group after 3, 6, and 9 months, but the FPG level was higher in the MSC-treated group than in the control group after a 12-month follow-up period. Moreover, the difference was not statistically significant with 3-, 6-, 9-, and 12-month follow-up as (MD = 0.74, 95% CI -0.54 to 2.02, *P*-value = 0.258), (MD = 0.20, 95% CI -0.34 to 0.73, *P*-value = 0.471), (MD = 0.02, 95% CI -0.52 to 0.57, *P*-value< = 0.932), and (MD = -1.11, 95% CI -3.10 to 0.88, *P*-value = 0.273), respectively, while in n-RCT the FPG in the MSC-treated group showed a significant decrease from its baseline level to that at the 6-month follow-up period (MD = 0.93, 95% CI 0.14 to 1.72, *P*-value = 0.021) (**Table 7**).

**Figure 5:**
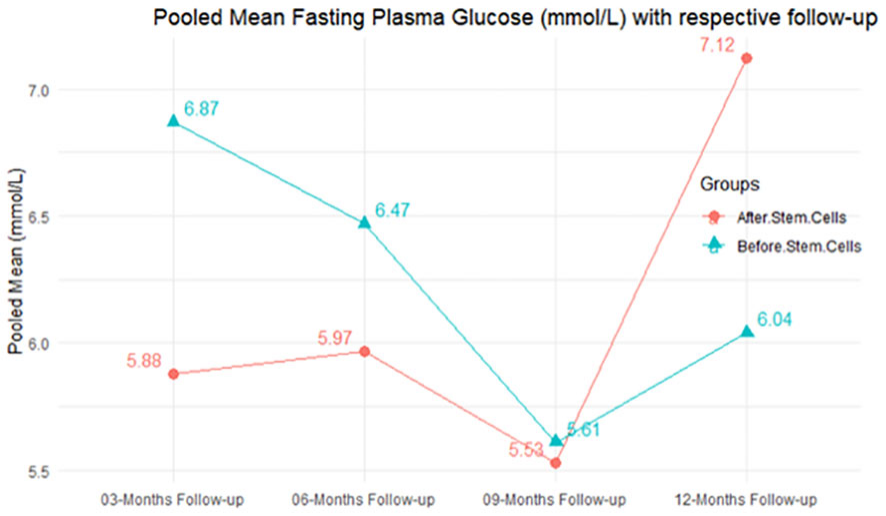
Pooled mean fasting plasma glucose (mmol/L) with respective follow-up periods.

The observed mean differences (MDs) in all included studies along with 3-, 6-, and 12-month follow-up were positive, which had indicated a decrease in fasting plasma glucose due to stem cell therapy, while the observed mean differences (MDs) in one study with 9-month follow-up were negative, which had indicated an increase in fasting plasma glucose due to stem cell therapy. The overall effect size for 3, 6, and 12 months was non-significant at *P*-value > 0.05. The results are shown in **Table 5**. The pooled mean of fasting plasma glucose computed from all studies with 3-, 6-, and 9-month follow-up showed a decrease in the levels of FPG due to stem cell transplantation, but at 12-month follow-up the levels of fasting plasma glucose showed an increase (see the graph/**Figure 5**).

### Effects of stem cell transplantation on C-peptide (ng/mL)

According to the forest plots shown in **Table 6**, the overall effect size evaluated with difference (MD) was disclosed when comparing the administration of the MSCs and the control group, which had shown a considerably reduced level of C-peptide at 5% level of significance in 3-, 6-, and 12-month follow-up as (MD: -0.12, 95% CI: -0.34 to 0.11, *P*-value: 0.0.300 > 0.05, *I*^2^: 48.39%), (MD: -0.03, 95% CI: -0.52 to 0.45, *P-*value: 0.895 > 0.05, *I*^2^: 90.66%), and (MD: -0.02, 95% CI: -0.07 to 0.02, *P-*value: 0.231 > 0.05, *I*^2^: 34.51%), respectively.

In RCT, we found that the level of C-peptide was increased in the MSC-treated group than in the control group after 3 and 12 months, but the C-peptide level was lower in the MSC-treated group than in the control group after a 6-month follow-up period. The difference was statistically non-significant with 3-, 6-, 9-, and 12-month follow-up as (MD = -1.11, 95% CI -3.10 to 0.88, *P*-value = 0.273), (MD = -0.05, 95% CI -0.33 to 0.22, *P*-value = 0.712), (MD = 0.06, 95% CI -0.22 to 0.33, *P*-value = 0.690), and (MD = -0.02, 95% CI -0.06 to 0.02, *P*-value = 0.250), respectively, while in n-RCT the C-peptide in the MSC-treated group showed an increase from its baseline level and at 3- and 6-month follow-up period (MD = -0.27, 95% CI -0.40 to -0.14, *P*-value< 0.001 and (MD = -0.01, 95% CI: -1.12 to 1.11, *P*-value = 0.991) (**Table 7**).

**Figure 6:**
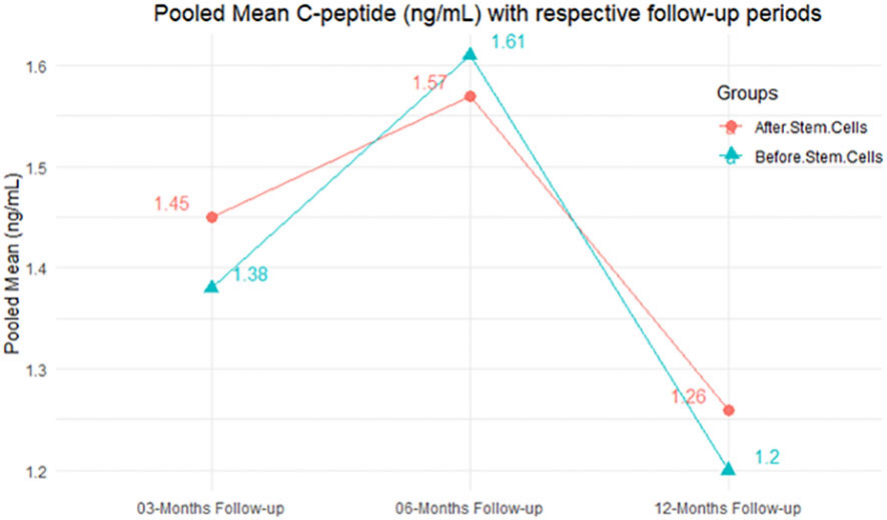
Pooled mean C-peptide (ng/mL) with respective follow-up periods.

The observed mean differences (MDs) in all included studies along with 3-, 6-, and 12-month follow-up were negative (52.63%), which had indicated a minor increase in C-peptide due to MSC transplantation, while 47.37% showed a positive response. From the graph/**Figure 6**, the pattern of the levels of C-peptide was random as observed at 3-, 6-, and 12-month follow-up. As all results of the comparison between stem cell therapy and control group for C-peptide were non-significant, there is a need, therefore, to conduct more studies with a long follow-up.

### Heterogeneity

Cochran’s *Q*-test and *I*^2^ statistic were applied to measure the heterogeneity of the true outcome of the following parameters: HbA1c, insulin requirement, fasting blood glucose, fasting plasma glucose, and C-peptide with 3-, 6-, 9-, and 12-month follow-ups. According to the Q-test, the true outcomes had appeared to be heterogeneous significantly for HbA1c with 6-, 9-, and 12-month follow-up as (*Q*-test: 12.547, *P*-value: 0.049< 0.05, tau-square: 0.1184, *I*^2^: 58.33%), (*Q*-test: 58.590, *P*-value< 0.001, tau-square: 0.0233, *I*^2^: 84.84%), (*Q*-test: 41.073, *P*-value< 0.001, tau-square: 3.2342, *I*^2^: 96.73%), and (*Q*-test: 25.908, *P*-value< 0.001, tau-square: 0.3551, *I*^2^: 87.33%), respectively (shown in **Table 2**). Similar results for other parameters can be found in **Tables 2**–**6**. The random-effect model was implemented for significance of heterogeneous true outcomes.

### Publication bias assessment

The publication bias is estimated through funnel plots and Begg’s and Egger’s regression tests for each forest plot of the following parameters: HbA1c, insulin, fasting blood glucose, fasting plasma glucose, and C-peptide with 3-, 6-, 9-, and 12-month follow-up. The empirical estimation of publication bias was indicated as non-significant bias at 5% level of significance for HbA1c in all 3-, 6-, 9-, and 12-month follow-up as (Begg and Mazumdar test, *P*-value: 0.733 > 0.05 and Egger’s regression *P*-value: 0.568 > 0.05), (Begg and Mazumdar test, *P-*value: 0.156 > 0.05 and Egger’s regression *P*-value: 0.091 > 0.05), (Begg and Mazumdar test, *P*-value: 0.333 > 0.05 and Egger’s regression *P*-value: 0.007< 0.05), and (Begg and Mazumdar test, *P*-value: 0.233 > 0.05 and Egger’s regression *P-*value: 0.001< 0.05), respectively. Similar results for publication bias about the parameters insulin requirement, FPG, FBG, and C-peptide can be found in **Tables 3**–**6**.

### Sensitivity analysis

In the present meta-analysis, sensitivity analysis was conducted to evaluate the impact of key methodological decisions on the synthesized effect estimates and associated uncertainty measures. Specifically, we explored the effects of alternative statistical models (e.g., fixed-effects vs. random-effects models), inclusion/exclusion of studies based on specific criteria (e.g., sample size, study quality), and variations in data synthesis techniques. Through this rigorous examination, we aimed to ascertain the robustness of our findings against potential sources of bias and heterogeneity inherent in meta-analytic research. By identifying influential studies, assessing the sensitivity of results to methodological assumptions, and exploring the consistency of conclusions across different analytical approaches, the sensitivity analysis provides valuable insights into the reliability and generalizability of our study findings.

**Figure 7:**
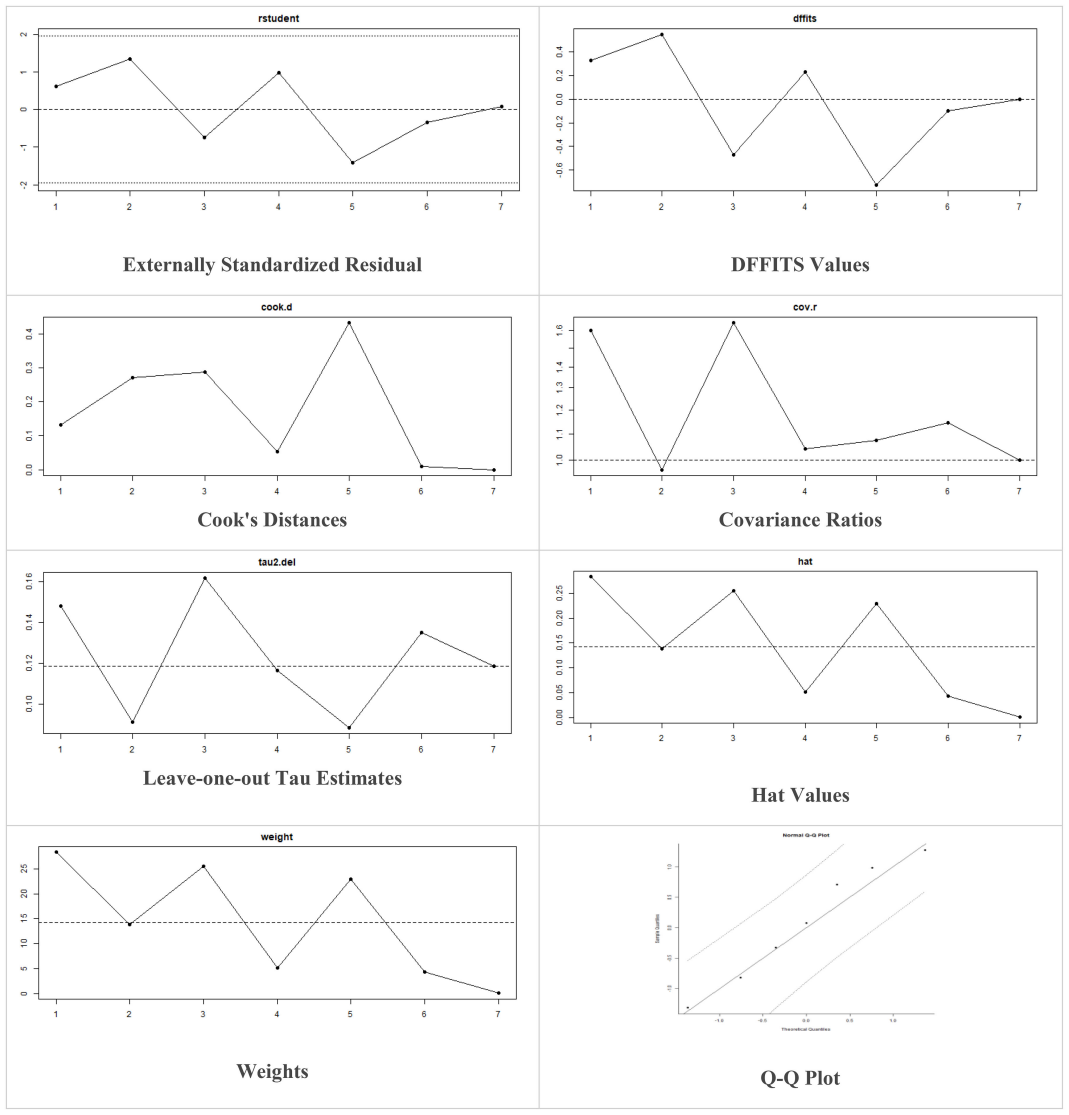
Plots of Sensitivity Analysis for HbA1c (%).

**Figure 8:**
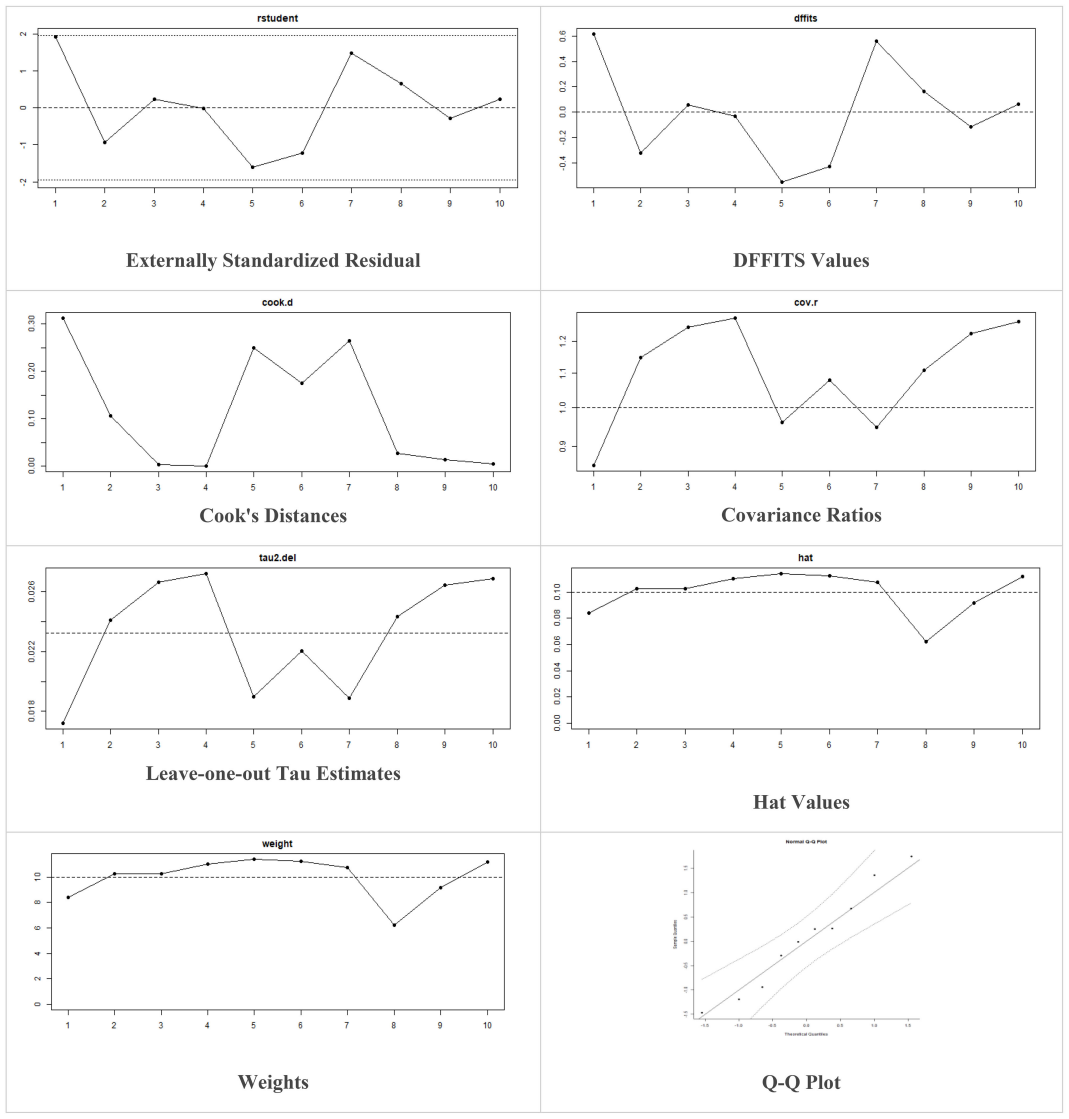
Plots of Sensitivity Analysis Tests for Insulin requirement (IU/kg/day).

**Figure 9:**
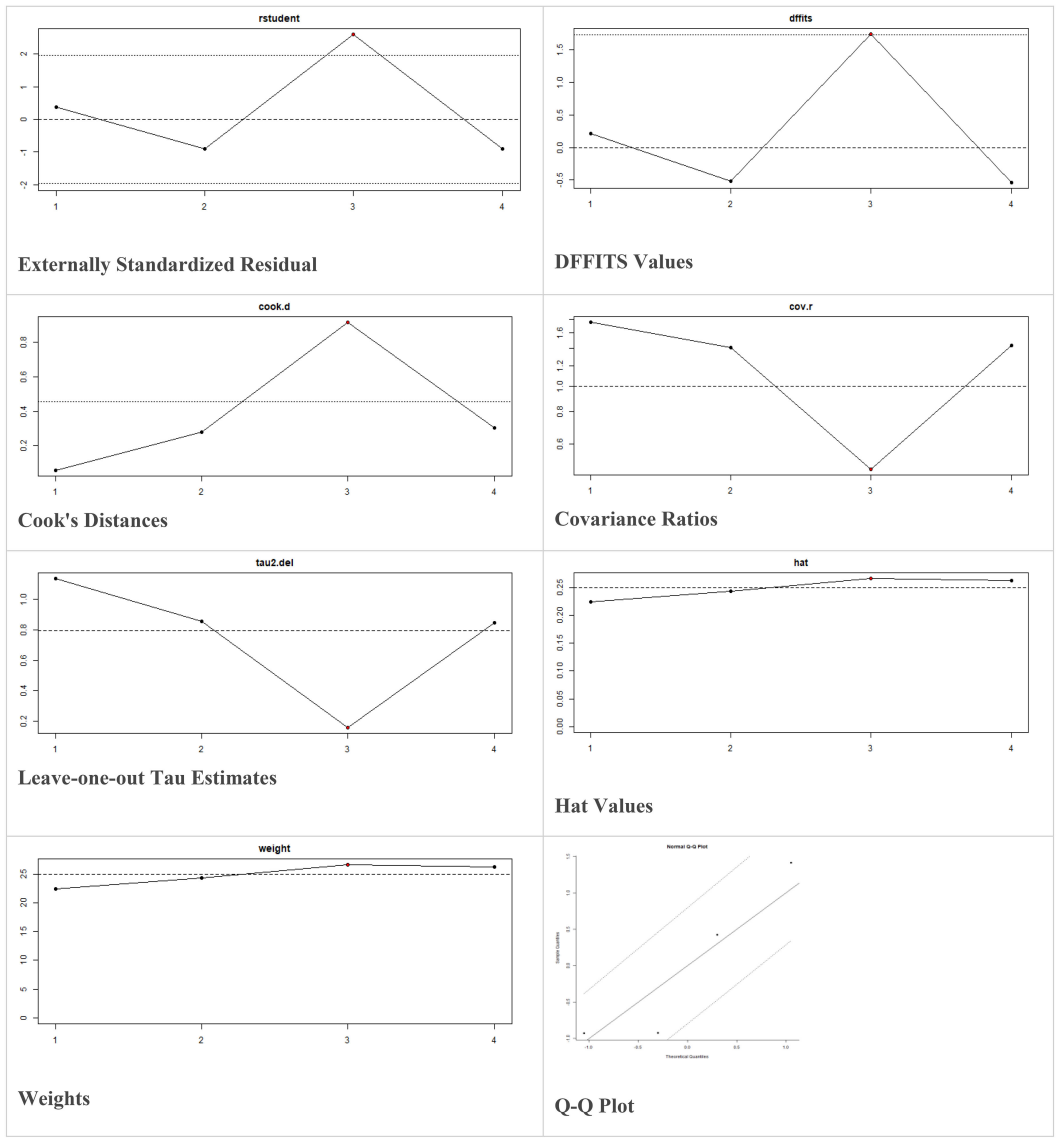
Plots of Sensitivity Analysis Tests for FPG (mmol/L).

**Figure 10:**
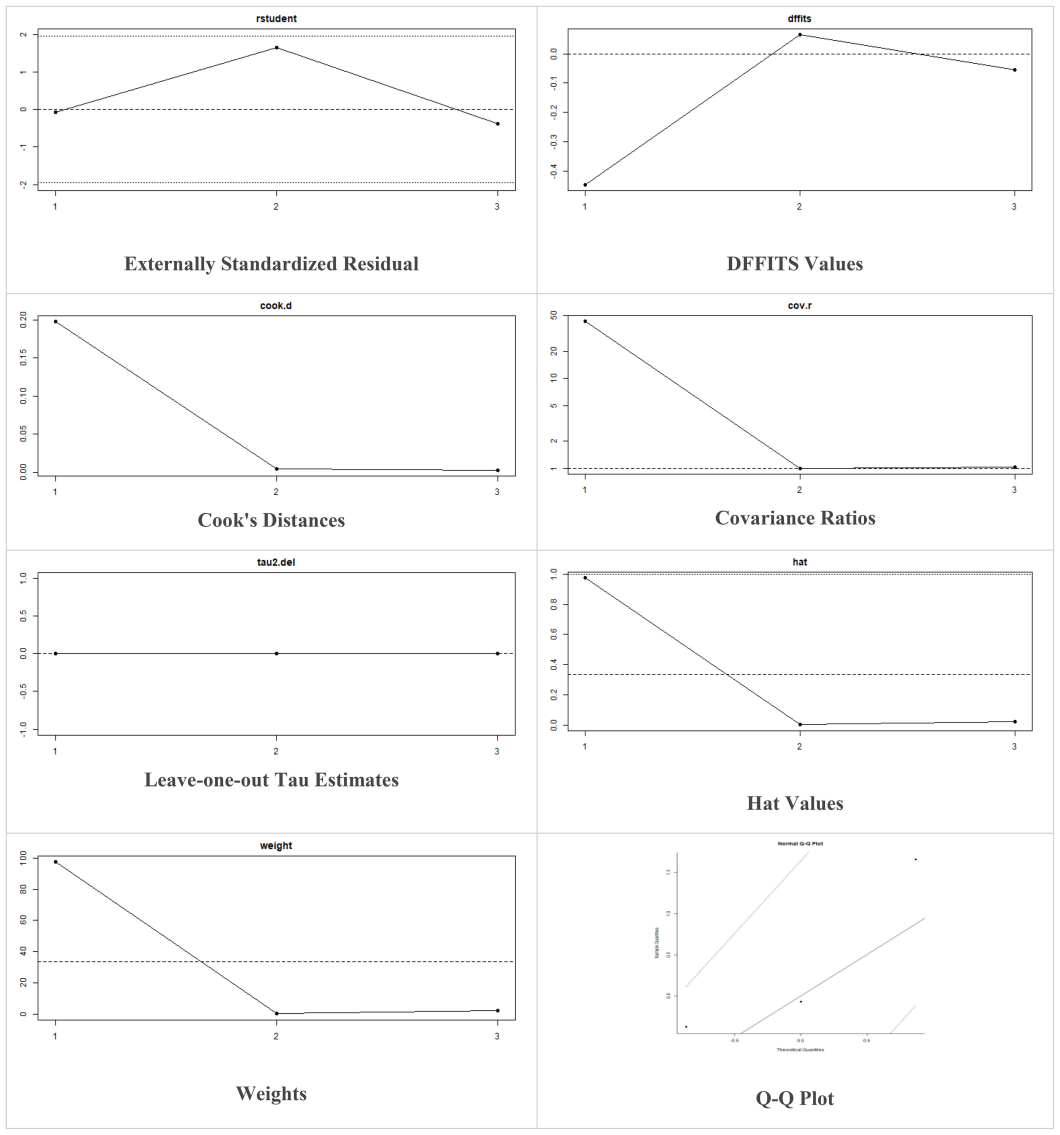
Plots of Sensitivity Analysis Tests for FBG (mmol/L).

**Figure 11:**
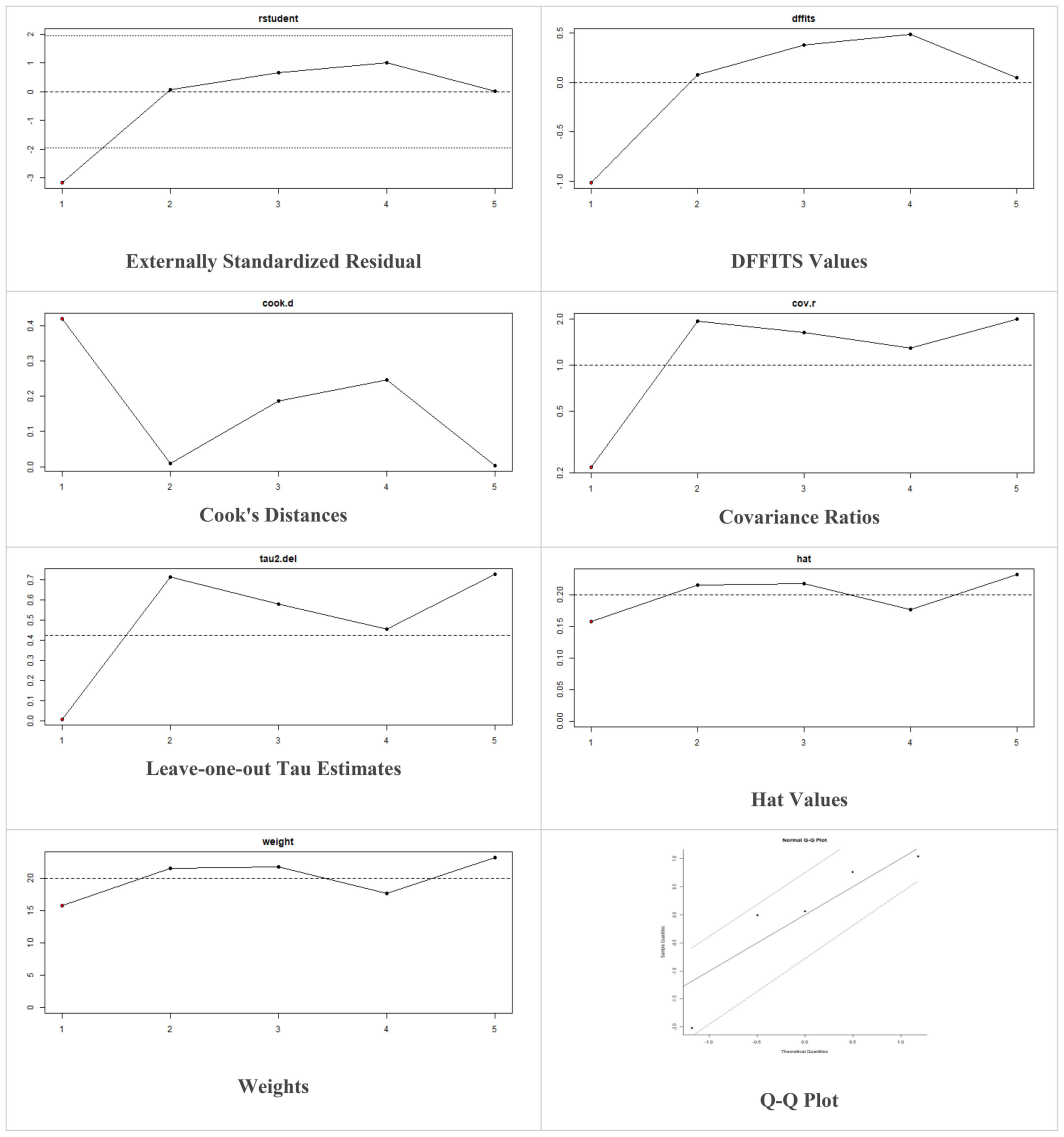
Plots of Sensitivity Analysis Tests for C-peptide (ng/mL).

Multiple methods including the externally standardized residuals, DFFITS values, Cook’s distances, covariances ratios, leave-one-out tau estimates, Hat values, and weights were applied and examined in instances that their residuals of fasting plasma glucose fall out of the control limits (**Figure 7**) due to a previously conducted study (37). After excluding this study, a similar approach was iteratively repeated and excluded (15) to minimize the potential risk of bias. The revised results for FPG are shown in **Table 5**. The plots of these sensitivity analyses for HbA1c, insulin requirement, FPG, FBG, and C-peptide are presented in **Figures 7**–**11**.

### MSC transplantation safety and adverse events

To clearly differentiate between potential complications caused by the intervention, the fundamental complications of T1DM and insulin therapy must be identified. Compliance to Good Clinical Practice (GCP) guidelines, including randomization and the inclusion of a control group, is recommended to facilitate this. However, the majority of clinical trials investigating stem cell transplantation for the treatment of T1DM have been of poor quality, with many lacking a control group or randomization to allow for comparisons of outcomes and adverse events. Consequently, there have been conflicting judgments regarding the side effects of stem cell therapy in these trials. Hypoglycemia was excluded from consideration as an adverse event, as it can happen due to insulin therapy and autoimmune disorders of the thyroid in individuals with T1DM without any intervention.

Minor hypoglycemic episodes were mentioned in three studies (15, 37, 38), but these episodes were not classified as severe. Nausea and vomiting were mentioned in three studies, with (38) not specifying the number of patients affected, while (15) and (30) reported one patient each. Bhansali et al. (15) also reported hemorrhage at the injection puncture site in one patient, a drop in hemoglobin level in two patients, and a self-limiting upper respiratory tract infection in one patient. Mild fever was reported in three out of 22 T2DM patients by Liu et al. (17). There were no serious or persistent adverse reactions or legacy effects observed during the follow-up timespan, indicating that MSCs are reasonably safe in the treatment of DM.

## Discussion

This meta-analysis provides quick insights about MSC transplantation along with their statistical significance (*P*-value< 0.05) and was associated with improvements in both T1DM and T2DM. The absence of observed adverse effects in the patients suggests that MSC transplantation may be a safe and promising approach to improve glucose metabolism in individuals with T1DM and T2DM. The data confirms the use of MSC transplantation as an effectual diabetes treatment. The significant reduction in fasting blood glucose, plasma blood glucose, and HbA1c levels at baseline suggests that MSC therapy can improve blood glucose regulation in diabetic patients. The use of FBG and PBG as diabetes diagnostic criteria, as well as HbA1c levels as a measure of diabetes control, supports the conclusion that MSC transplantation has a therapeutic effect on blood glucose regulation in DM patients.

The meta-analysis results indicate a slight but non-significant rise in fasting C-peptide levels in the group that received MSC transplantation (*P*-value > 0.05). The escalation in F-CP level decreased as the duration of follow-up increased. This increase in F-CP indicates an improvement in insulin secretion by the pancreatic islet cells, implying that MSC transplantation has a beneficial effect on insulin secretion. The elevated insulin secretion could be due to either a rise in the number of insulin-secreting cells or an improved performance in the function of the remaining β cells. These findings suggest that MSC transplantation has potential as a treatment for diabetes, but further research with longer follow-up periods is necessary to fully comprehend the underlying mechanisms and ensure its long-term safety and effectiveness.

Our findings revealed a substantial reduction in insulin demands following MSC therapy in patients with diabetes, which was consistent across all included studies with follow-up periods of 3, 6, 9, and 12 months. This decrease in insulin requirements was found to be statistically significant (*P*-value< 0.05). The observed efficacy of MSC therapy in reducing insulin requirements was retained at the end of most follow-up intervals. However, further studies with prolonged follow-up time points and complete data must confirm these findings. The cessation of insulin treatment is a crucial component in enhancing the overall quality of life of individuals with diabetes. In some studies, it was regarded as the primary outcome. A total of three patients in (34), three in (36), two in (28), six in (37), and five in (32) experienced an insulin-free period.

The findings of the meta-analysis indicate a potential improvement in the efficacy of stem cell transplantation for diabetes treatment from 3 to 12 months after transplantation. However, some of the trends were not statistically significant. The results suggest that the MSC transplantation group experienced improvement from 3-, 6-, 9-, and 12-month follow-up periods. However, to ensure the safety and efficacy profiles of SCT for diabetes treatment, long-term follow-up studies are necessary. Thus, there is a need to conduct more studies with extended follow-up periods to obtain a better understanding of the effects of SCT on diabetes. Moreover, further studies that will emphasize on clarifying the different follow-up phases or describing the primary outcomes related to the impact of SCT on diabetes morbidity and mortality are recommended.

## Conclusion

According to the analysis, MSCs have been demonstrated as a secure option for stem cell transplantation in diabetes mellitus. The short-term findings indicated that MSCs could help enhance blood glucose regulation; however, additional research is necessary to assess their long-term impacts. Across the 13 studies, no significant adverse reactions or occurrences of hypoglycemic events were detected in subjects who received MSCs treatment. This suggests that MSC transplantation can be regarded as a safe treatment option for DM.

### Strengths and limitations

The performance of MSC transplantation in the treatment of diabetes mellitus was analyzed in this systematic review and meta-analysis. The study searched numerous databases and trial registries from their establishment until February 2023. The study utilized a consensus approach to settle disputes, neutral supervision for data extraction, inclusion and exclusion criteria, top-notch impact illustration of original research studies findings on meta-analysis results, and confidence intervals for cumulative facts. Regardless of these advantages, the study had flaws, such as insufficient well-designed clinical trials with control groups, randomization, and blinding. Most of the SCT clinical trials in DM were single arm, leading to inconclusive results. Therefore, standardization and uniformity in the production, culture, and administration of MSCs in clinical trials are needed. In addition, the long-term safety and efficacy of MSC-based therapies have yet to be established, and larger sample sizes, more extended follow-up periods, and well-designed randomized controlled trials are needed to provide a comprehensive assessment of the benefits and risks of these treatments. It is also suggested that expressing daily insulin levels in units/kg/day instead of just customary units can provide a more standardized and comparable measure of the treatment’s effect. Furthermore, presenting findings in numeric form, rather than just figures, can increase the clarity and comprehensiveness of the results. In conclusion, while MSC transplantation shows promise in treating T1DM and T2DM, further research is necessary to fully understand its safety and efficacy and establish best practices for its use in clinical trials. Furthermore, limitations exist due to the lack of individual-level data required for subgroup analysis, such as age, gender, sickness status, and duration of disease history. The decision of selecting confounding factors was contingent upon data availability. The selection of confounding factors for inclusion in the study was determined subjectively, acknowledging inherent limitations. Complete elimination of potential interference from other factors was not feasible. Therefore, future research endeavors should encompass a broader scope, incorporating additional articles to further elucidate the collective impact of multiple factors on diabetes mellitus. Additionally, constraints in implementing alternative statistical methods such as *t*-tests, ANOVA, or regression analysis due to the lack of original research data and distribution details of variables pose challenges. However, the inability to conduct detailed subgroup analyses and the reliance on summarized data may limit the depth of insights and generalizability of findings.

## Data availability statement

The raw data supporting the conclusions of this article will be made available by the authors, without undue reservation.

## Author contributions

UH: Data curation, Formal analysis, Investigation, Methodology, Software, Visualization, Writing – original draft, Writing – review & editing. NK: Data curation, Formal analysis, Project administration, Supervision, Validation, Writing – review & editing. DG: Conceptualization, Funding acquisition, Project administration, Resources, Validation, Writing – review & editing. KA: Data curation, Investigation, Methodology, Software, Validation, Visualization, Writing – review & editing. SS: Data curation, Resources, Writing – review & editing. AU: Data curation, Resources, Writing – review & editing.
