# Supplementary material for: Meta-analysis shows that mesenchymal stem cell therapy can be a possible treatment for diabetes
Source: Front Endocrinol (Lausanne). 2024 May 10;15:1380443. doi: 10.3389/fendo.2024.1380443 (PMC11116613; doi:10.3389/fendo.2024.1380443)
Supplement: Supplementary file 1 [file DataSheet_1.docx]

**Appendix 1**

**Searches strategy**

We searched PubMed, Web of Science, PubMed, the Cochrane Library and Google Scholar using the following key words: (“mesenchymal stem cells, mesenchymal stromal cells, Wharton’s Jelly cells, progenitor cells, bone marrow” or “MSCs”) AND (“diabetes mellitus” or “hyperglycemia”) AND “diabetic kidney disease” AND (“English language” OR “Chinese language”). The retrieval time was from establishment of these databases to November 2023. The detailed search strategies are listed below.

**PubMed Syntax**

(("stem cells"[Title/Abstract] OR "progenitor cells"[Title/Abstract] OR "mesenchymal stem cells"[Title/Abstract] OR "bone marrow mononuclear cells"[Title/Abstract] OR "cell therapy"[Title/Abstract] OR "diabetes"[Title/Abstract] OR "hyperglycemia"[Title/Abstract]) AND ("stem cells"[MeSH Terms] OR ("stem"[All Fields] AND "cells"[All Fields]) OR "stem cells"[All Fields])) AND (clinicaltrial[Filter])) AND 2000/01/01:2023/11/15[dp]

PubMed #1 stem cells [Title/Abstract]

#2 progenitor cells [Title/Abstract])

#3 mesenchymal stem cells [Title/Abstract]

#4 bone marrow mononuclear cells [Title/Abstract]

#5 cell therapy [Title/Abstract]

#6 #1 OR #2 OR #3 OR #4 OR #5 OR #6

#7 diabetes [Title/Abstract]

#8 hyperglycemia [Title/Abstract]

#9 #7 OR #8

#10 #7 AND #9 filters: clinical trial

#11 #7 AND #9 filters: clinical trial

**Cochrane:**

The Cochrane Library

#1 (diabetes): ti, ab, kw OR (hyperglycemia):ti, ab, kw (Word variations have been searched)

#2 (stem cells): ti, ab, kw OR (mesenchymal stem cells):ti, ab, kw OR (bone marrow mononuclear cells):ti, ab, kw AND (cell therapy):ti, ab, kw (Word variations have been searched)

#3 (#2 (stem cells): ti, ab, kw OR (mesenchymal stem cells):ti, ab, kw (Word variations have been searched)

#4 #2 AND #3 AND #1 in Trials

**ClinicalTrials.gov**

“diabetes” AND “stem cells” OR “mesenchymal stem cells” | studies with Results

**Web of Science:**

#1 T1 = diabetes

#2 T1 = hyperglycemia

#3 T1 = mesenchymal stem cells

#4 T1 = mesenchymal stromal cells

#5 T1 = mesenchymal progenitor cells

#6 T1 = bone marrow stromal cells

#7 T1 = bone marrow derived stem cells

#8 T1 = Wharton’s Jelly cells

#9 #1 OR #2

#10 #3 OR #4 OR #5 OR #6 OR #7 OR #9

#11 #9 AND #10

#12 (#11) AND LANGUAGE: (English)
